# Associations of resting heart rate with incident dementia, cognition, and brain structure: a prospective cohort study of UK biobank

**DOI:** 10.1186/s13195-022-01088-3

**Published:** 2022-10-05

**Authors:** Yue-Ting Deng, Kevin Kuo, Bang-Sheng Wu, Ya-Nan Ou, Liu Yang, Ya-Ru Zhang, Shu-Yi Huang, Shi-Dong Chen, Yu Guo, Rui-Qi Zhang, Lan Tan, Qiang Dong, Jian-Feng Feng, Wei Cheng, Jin-Tai Yu

**Affiliations:** 1 Department of Neurology and Institute of Neurology, Huashan Hospital, State Key Laboratory of Medical Neurobiology and MOE Frontiers Center for Brain Science, Shanghai Medical College, Fudan University, 12th Wulumuqi Zhong Road, Shanghai, 200040 China; 2 National Center for Neurological Disorders, Shanghai, China; 3 Department of Neurology, Qingdao Municipal Hospital, Qingdao University, Qingdao, China; 4 Institute of Science and Technology for Brain-inspired Intelligence, Fudan University, Shanghai, China

**Keywords:** Cognitive impairment, Dementia, Alzheimer’s disease, Vascular dementia, Cardiovascular diseases, Resting heart rate

## Abstract

**Background:**

Resting heart rate (RHR) has been linked with an increased risk of dementia. However, evidence characterizing the associations of RHR with different dementia subtypes and their underlying mechanisms remains scarce. This study aims to investigate the relationships of RHR with different dementia types, cognitive function, and brain structural abnormalities.

**Methods:**

Three hundred thirty-nine thousand nine hundred one participants with no prior diagnosis of dementia from the UK biobank were analyzed. Cox regression and restricted cubic spline models examined the associations between RHR with all-cause dementia (ACD) and its major subtypes—Alzheimer’s disease (AD) and vascular dementia (VaD). Logistic regression models assessed the associations of RHR with cognitive function, and linear regression models estimated the associations with hippocampal subfield volume and white matter tract integrity indexed by magnetic resonance imaging data.

**Results:**

During an average of 3148 (± 941.08) days of follow-up, 4177 individuals were diagnosed with dementia, including 2354 AD and 989 VaD cases. RHR ≥ 80bpm was associated with ACD (HR: 1.18, 95% CI: 1.08–1.28, *P* < 0.001) and VaD (HR: 1.29, 95% CI: 1.08–1.54, *P* = 0.005) but not AD in multi-adjusted models. A 10-bpm increment of RHR demonstrated non-linear effects in VaD, consisting of J-shape relationships. Several heterogeneities were indicated in stratified analysis, in which RHR measures only showed associations with dementia incidents in relatively younger populations (age ≤ 65) and females. Apart from dementia analysis, elevated RHR was associated with worsening performance in fluid intelligence and reaction time of cognitive tasks, decreased hippocampal subfields volume, and poor white matter tract integrity.

**Conclusions:**

RHR is associated with increased risks of ACD and VaD but also presented with few heterogeneities across different sex and age groups. Elevated RHR also appears to have deleterious effects on cognitive function and is distinctively associated with volume reduction in hippocampal subfields and impaired white matter tract integrity.

**Supplementary Information:**

The online version contains supplementary material available at 10.1186/s13195-022-01088-3.

## Background

Dementia, characterized by a progressive deterioration in cognitive capacities, is one of the leading causes of morbidity and mortality in elderly individuals. Approximately 47 million were affected by dementia on a global scale, and this number is anticipated to triple in 2050 with a transitioning demographics of an aging population [1].

Owning to the absence of disease-modifying treatment, the current therapeutics strategy for dementia has been fundamentally centered on prevention. Aggressive management of cardiovascular risk factors, including diabetes, obesity, smoking, and hypertension, has been mightily advocated as published works show their associations with not only cardiovascular diseases (CVD) but also different dementia types [2]. Specific CVD subjecting to similar epidemiological profiles, such as atrial fibrillation and heart failure, has also been identified as at-risk conditions that increase dementia incidences [3, 4]. Still, these factors may only recapitulate partial aspects of dementia’s development, and their presence may have indicated a long-standing detriment that remains undetected. The challenge, therefore, is to widen the window of interventional opportunities by identifying novel targets preceding the emergence of relevant factors.

Resting heart rate (RHR), a crucial determinant for myocardial performance and blood perfusion, has been established as a prognostic and modifiable marker for CVD [5]. In recent years, there has been growing recognition of the predictivity of RHR beyond CVD, particularly for dementia. Several prospective studies have demonstrated that elevated RHR is associated with increased risks of cognitive decline and dementia in long-term follow-up [6, 7]. However, their relationships have courted controversy as null associations are yielded [8]. Furthermore, it is unclear whether RHR is distinctively associated with different dementia subtypes because the subsistent approaches have been constrained to all-cause dementia (ACD).

In terms of the neurobiological underpinnings between RHR and dementia, no definite conclusion has been given due to the nature of prima facie evidence. Yet, studies have unveiled brain structural abnormalities, including subcortical lesions, silent infarcts, and white matter hyperintensity volume, in the elderly with elevated RHR, complementing its underlying role in promoting dementia [9, 10].

Given the ambiguity, a prospective investigation embedded with a large population size may overcome the existing limitations of statistical power and sample representation. Additionally, the further incorporation of dementia subtypes and imaging may also provide greater pellucidity on the effects of RHR. Herein, we leverage the UK biobank’s magnitude participants and longitudinal follow-up to examine the associations of RHR with ACD and its subtype—Alzheimer’s disease (AD) and vascular dementia (VaD)—to elucidate the potential linkages extensively. We also extrapolate that individuals with elevated RHR will carry greater risks of structural alterations in the hippocampus based on the indicative evidence from preliminary investigations.

## Method

### Study population and design

The UK Biobank is a large population-based prospective cohort, constituting 502,493 individuals who were 40–69 years of age at the time of recruitment between 2006 and 2010 [11]. At enrollment, participants provided electronically signed consent and were invited to attend 1 of the 22 centers for baseline assessments. The extensive information was collected via touchscreen questionnaires, interviews, physical measures, health records, and biological samples. A detailed description of the cohort and protocol can be found on the official webpage (http://www.ukbiobank.ac.uk/wpcontent/uploads/2011/11/UK-Biobank-Protocol.pdf).

Individuals with confirmed diagnoses of dementia at baseline and missing information on RHR and Apolipoprotein E (*APOE* 4) status were excluded. In total, there were 339,901 dementia-free participants eligible for the analyses. The illustration of the study workflow is presented in Supplementary Materials 1.

### Measurement of RHR

The primary study exposure was RHR, and its measurement was based on the pre-defined standard operating procedure at the initial study visit. Briefly, the participant was requested to sit in a relaxed position, with their lower limbs parallel to each other, feet flat on the floor, and their arm on the desktop. Any restrictive clothing was either loosened or removed, and staff members were informed to avoid verbal contact. The measurement was taken from the mid-point circumference of the left arm, whereas the right arm was used in case of an infeasible scenario (shunt, amputee, mastectomy, and axillary clearance). The Omron 705 IT electronic blood pressure monitor (OMRON Healthcare Europe B.V. Kruisweg 577 2132 NA Hoofddorp) was used as the primary device, and the readings of blood pressure and pulse rate were displayed in Omron’s monitor and auto-populated to the electronic records. The heart rate readings were then utilized in the analysis.

### Ascertainment of covariates

Age and gender were attained during the first study visits. Education was self-reported with six response categories, and it was further recoded into an ordinal scale from 0 to 10 based on the years of full-time formal education(0: 0 years, 1: 1–4 years, 2: 5–9 years, 3: 10–11 years, 4: 12–13 years, 5: 14–15 years, 6: 16–17 years, 7: 18–19 years, 8: 20–21 years, 9: 22–23 years, 10: > 24 years of education) [12]. *APOE* 4 status was determined by the presence of carriers through the genetic database. Tobacco use was self-reported by participants and categorized into never, former, and current smokers. Body mass index (BMI) was calculated with the weight of the individual in kilograms divided by the square of the individual’s standing height in meters. Metabolic equivalent minutes per week (MET-min/wk) was used to represent the amount of total physical activities, which was derived from the modified version of the international physical activity questionnaire [13]. Diabetes mellitus and hypertension were determined either by the self-reported response or confirmed diagnosis from a doctor. CVDs, including heart failure, atrial fibrillation, ischemic heart disease, and cerebrovascular diseases, were ascertained by self-reported conditions, hospital records, and death registry. For heart rate modifying medications, the following self-reported usage was considered: beta-blockers, digoxin, and non-dihydropyridine calcium channel blockers. Serum total cholesterol was measured at baseline.

### Ascertainment of incident dementia

The primary outcomes in this study were ACD and its major components—AD and VaD. The ascertainment of dementia types used the hospital inpatient records on admissions and diagnoses from Hospital Episodes Statistics for England, Scottish Morbidity Record data for Scotland, and the Patient Episode Database for Wales. Death register data were also collected from the National Health Service Digital for England and Wales and the Information and Statistics Division for Scotland to detect additional cases. We defined outcomes in accordance with International Classification of Diseases (ICD) 9 and 10 codes: ACD (A81.0, F00, F01, F02, F03, F05.1, F10.6, G31.0, G31.1, and G31.8), AD (331.0, F00, and G30), and VaD (290.4, F01, and I67.3). Read codes versions 2 and 3 were also used for primary care records.

### Cognitive function

Cognitive tasks were bespoke to UK biobank and evaluated at baseline and imaging visit through touch-screen, covering different aspects of cognitive capacity. Further information is described on the website. In this study, we conducted screening across 5 cognitive tasks, including fluid intelligence, reaction time, numeric memory, prospective memory, and pairs matching. We then focused on fluid intelligence and reaction time tasks as they were administrated throughout two visits and presented with statistical significance in initial analysis (Supplementary Materials 2). Cognitive decline as dichotomous variables were defined if the score of fluid intelligence was decreased by at least 1 point and reaction time slowed by at least 100 milliseconds in the follow-up assessment. Cognitive decline as continuous variables were defined by the absolute changes in cognitive scores between baseline and the follow-up assessment.

### Hippocampal subfield imaging and diffusion-tensor imaging data

MRI imaging protocols were designed by the UK Biobank Imaging Working Group (http://www.ukbiobank.ac.uk/expert-working-groups). Details regarding image acquisition and processing can be assessed in the previously published article [14]. Briefly, MRI data were acquired in a single Siemens Skyra 3T scanner with a 32-channel radiofrequency receive head coil located at the recruitment center at Stockport. The T1-weighted scans were obtained using three-dimensional magnetization-prepared rapid gradient-echo (resolution 1 mm^3^ isotropic voxels) and analyzed with the Functional Magnetic Resonance Imaging of the Brain Software Library (http://fsl.fmrib.ox.ac.uk/fsl). The volumes of the whole hippocampus and its subfield regions (i.e., CA1, CA3, CA4, fimbria, granule layer-molecular layer-dentate gyrus boundary (GC-ML-DG), hippocampal-amygdaloid transition area (HATA), hippocampal tail, hippocampal fissure, paralaminar nucleus, subiculum, and presubiculum) were subsequently generated (*N* = 23,714). Fractional anisotropy (FA) values of 27 white matter tracts were imaged using diffusion tensor imaging (DTI), where higher FA values suggest better brain integrity. Outlier data points, defined as being further than ± 4 SD from the mean, were excluded (< 1% of values).

### Statistical analysis

The baseline characteristics were presented as numbers (percentages) for categorical variables and as means (standard deviation) for continuous variables and compared with one-way analysis of variance and chi-square test. The multiple imputations by chained equation were applied for missing covariates to avoid potential bias using the MICE package in R.

RHR was categorized into < 60, 60–69 (reference), 70–79, and ≥ 80 bpm according to prior studies and also treated as continuous variable (per 10 bpm increment) for evaluation. We applied Cox proportional hazards regression models to examine the longitudinal associations of RHR with dementia and its subtypes. Model 1 minimally adjusted for age, sex, education, and *APOE* 4 Status; model 2 furtherly adjusted for behavioral risk factors (smoking, physical activity, and BMI); and model 3 completely adjusted for the above-mentioned alongside vascular risk factors (diabetes, hypertension, and total cholesterol), CVDs (ischemic heart disease, atrial fibrillation, heart failure, and cerebrovascular diseases), and medications. The results were presented in Hazard ratios (HR) and 95% confidence intervals (95% CI). For continuous RHR measures, the non-linearity was tested using cubic splines with three knots set as 10th, 50th, and 90th percentiles to facilitate comparison against the models with linear terms. Moreover, since *APOE* genotype data was missing in about six thousand participants, we conducted sensitivity analyses with *APOE* status unadjusted. Next, we performed two sensitivity analyses to consolidate the robustness of the results by excluding participants with any outcome events that occurred within the first 5 years of follow-up and by excluding participants who developed at least one of the CVDs at baseline to minimize confounding effects. Subsequently, interaction terms for age and sex were utilized to estimate whether the strata effect existed (*P* < 0.1). After that, we conducted two subgroup analyses through stratification of sex and age (i.e., ≤ 65 years and > 65 years). Furthermore, we evaluated whether there is an interaction between RHR and heart rate reducing medication by interaction terms and stratified analyses.

Cognitive decline as dichotomous variables were analyzed by logistic regression models and cognitive decline as continuous variables and imaging data by linear regression models with identical adjustment of Cox regression analysis and reported as the Odds ratios (OR). False discovery rate (FDR) was used to correct *P* values [15].

R software version 4.1.0 and GraphPad Prism version 8.00 (GraphPad Software, San Diego, CA) were used for statistical analyses and figure preparation. The significance threshold was set at a *P* < 0.05 (two-sided).

## Results

### Basic characteristics of participants

Of the 339,901 participants without the dementia incidents at baseline, the mean age was 57.29 (± 7.92) years, 42% were women, and 28% were *APOE 4* allele carriers. The higher RHR groups were more likely to be current smokers, less educated, less physically active, and to have higher BMI, TC levels, and fewer usages of heart rate reduction medications. There were no significant differences in the prevalence of CVDs between RHR groups. However, there were higher proportions of hypertension and diabetes in higher RHR groups (Table 1).

**Table 1:** Baseline characteristics of study participants by incident dementia status

	Overall	Non-dementia	Incident dementia	***P***
***N***	339901	335724	4177	
**Age (mean (SD))**	57.29 (7.92)	57.20 (7.91)	64.49 (4.51)	< 0.001
**Sex, female (%)**	156895 (46.2)	154670 (46.1)	2225 (53.3)	< 0.001
**Education (mean (SD))**	4.81 (2.42)	4.82 (2.42)	4.12 (2.35)	< 0.001
***APOE*** **4 carrier (%)**	97298 (28.6)	95020 (28.3)	2278 (54.5)	< 0.001
**Physical activity (mean (SD))**	2268.38 (2307.41)	2269.71 (2307.79)	2162.01 (2274.12)	0.003
**BMI (mean (SD))**	27.52 (4.79)	27.51 (4.78)	27.76 (4.94)	0.001
**Total cholesterol (mean (SD))**	5.70 (1.15)	5.71 (1.15)	5.52 (1.29)	< 0.001
**Smoking status (%)**				< 0.001
Never smoked	181967 (53.5)	180060 (53.6)	1907 (45.7)	
Former smoker	122727 (36.1)	120877 (36.0)	1850 (44.3)	
Current smoker	35207 (10.4)	34787 (10.4)	420 (10.1)	
**Hypertension (%)**	167914 (49.4)	165376 (49.3)	2538 (60.8)	< 0.001
**Diabetes (%)**	17473 (5.1)	16898 (5.0)	575 (13.8)	< 0.001
**CVDs (%)**	22391 (6.6)	21618 (6.4)	773 (18.5)	< 0.001
**Heart rate reducing medication (%)**	25921 (7.6)	25226 (7.5)	695 (16.6)	< 0.001
**Resting heart rate (mean (SD))**	69.61 (11.74)	69.60 (11.72)	70.46 (12.64)	< 0.001
**Resting heart rate (%)**				< 0.001
< 60 bpm	63711 (18.7)	62927 (18.7)	784 (18.8)	
60~70 bpm	118650 (34.9)	117309 (34.9)	1341 (32.1)	
70~80 bpm	95571 (28.1)	94427 (28.1)	1144 (27.4)	
> 80 bpm	61969 (18.2)	61061 (18.2)	908 (21.7)	

Shown are numbers (%) or mean (SD). *P*-values are derived using either Student’s *t*-test or chi-square test

*Abbreviations*: *APOE 4* apolipoprotein, *BMI* body mass index, *CVDs* cardiovascular diseases

### Associations between RHR and dementia outcomes

**Fig. 1:**
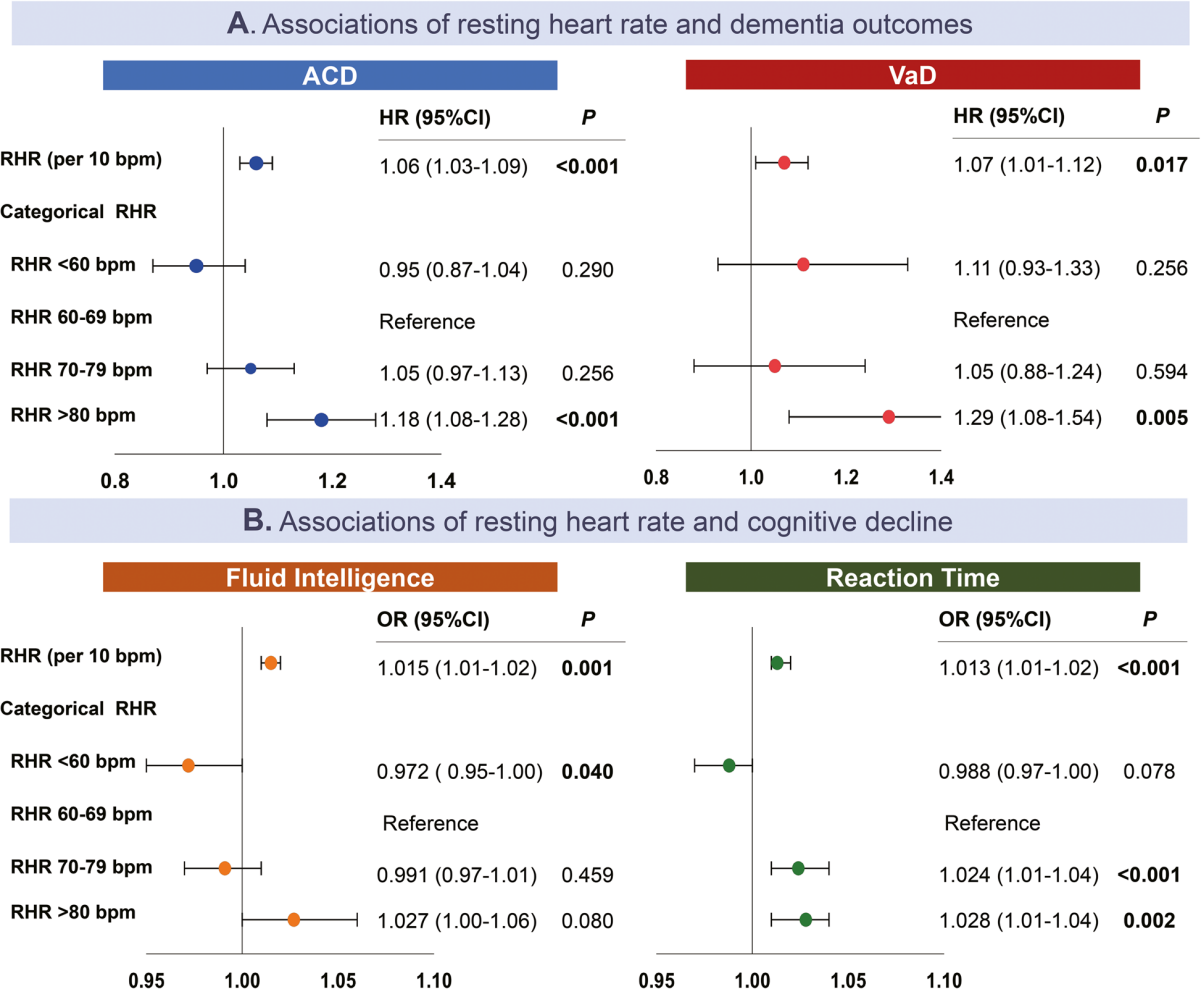
Associations of resting heart rate with dementia outcomes and cognitive decline. **A**, **B** Illustration of multiple-adjusted hazard ratios of RHR with dementia outcomes and cognitive decline. RHR, resting heart rate; HR, hazard ratio; OR, odds ratio; 95% CI, 95% confidence interval

**Fig. 2:**
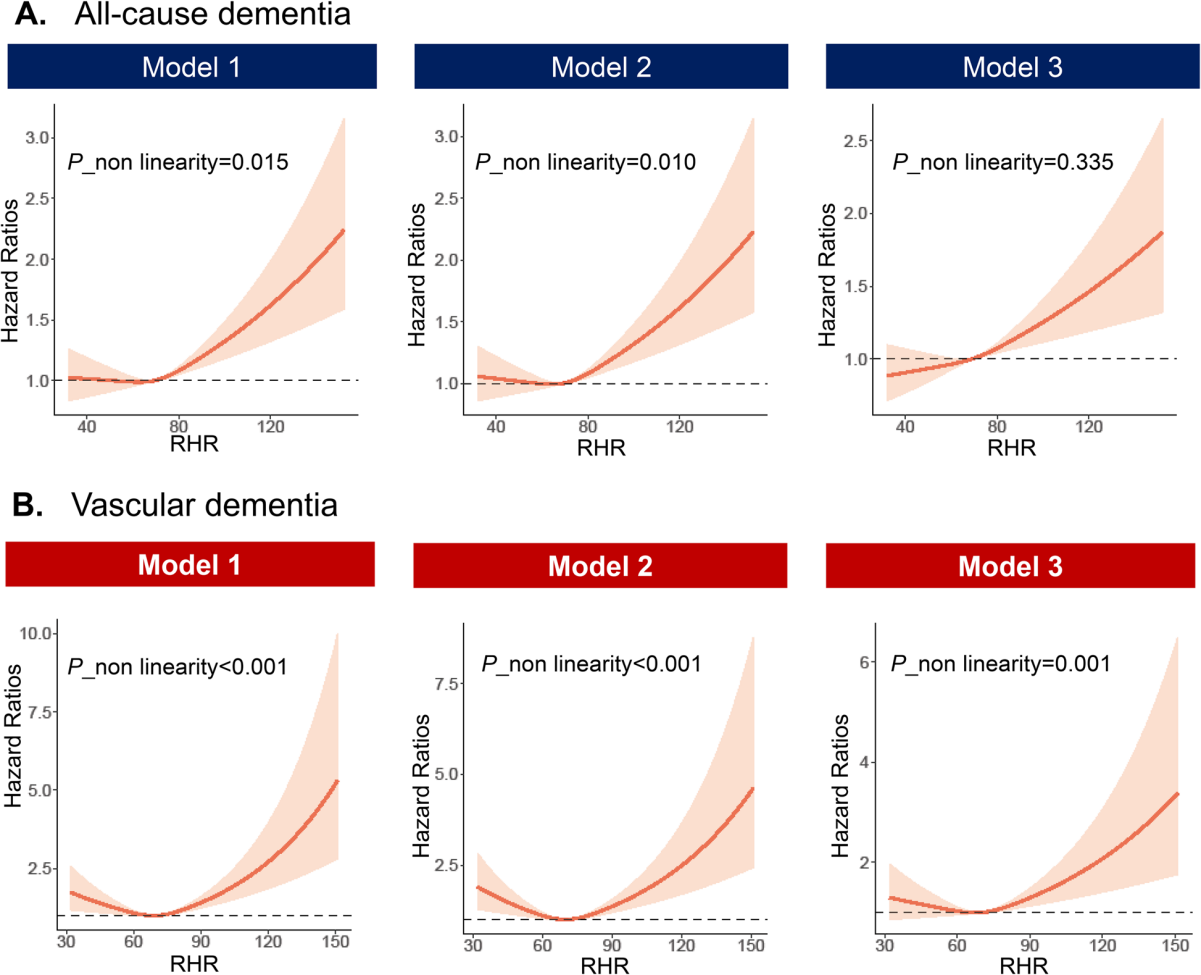
Testing for non-linearity of resting heart rate in dementia analyses. **A**, **B** Non-linearity of continuous RHR with the risk of all-cause dementia and vascular dementia

During an average of 3148 (± 941.08) days of follow-up, there were 4177 individuals diagnosed with dementia, which consists of 2354 AD and 989 VaD cases. In the fully adjusted models, participants with RHR ≥ 80 bpm demonstrated increased risks of developing ACD (HR: 1.18, 95% CI: 1.08–1.28, *P* < 0.001) and VaD (HR: 1.29, 95% CI: 1.08–1.54, *P* = 0.005) compared to those with RHR 60–69 bpm (Fig. 1). A 10-bpm increment of RHR was associated with a 6% greater hazard of ACD (HR: 1.06, 95% CI: 1.03–1.09, *P* < 0.001) and VaD (HR: 1.07, 95% CI: 1.01–1.12, *P* = 0.017). In models not adjusting for *APOE* 4 status, the magnitude and direction of RHR’s associations with dementia are sustained (Supplementary Materials 3). Using cubic spinal cubes, we found the patterns of non-linearity appeared to be more generalized in VaD, consisting of J-shape associations (Supplementary Materials 4). However, there were no non-linear effects of RHR in model 3 for ACD (Fig. 2).

In the sensitivity analysis, the exclusion of individuals with CVD at baseline did not result in attenuation of statistical significances for ACD and VaD. When removing dementia incidents occurring within 5 years, the associations between RHR and ACD remained robust (HR: 1.06, 95% CI: 1.03–1.09, *P* < 0.001), whereas the associations with VaD became insignificant (Supplementary Materials 5). As indicated by the interaction effects by age and sex (Supplementary Materials 5), we performed age- and sex-restricted models and identified several differences across different subgroups. Interestingly, for participants > 65 years, during an average of 8.90 ± 2.6 years follow-up, no associations were identified between RHR and incidents of any dementia types, except for participants with RHR > 80 bpm. However, for participants ≤ 65 years, a 10-bpm increment of RHR was associated with 4% and 9% greater risks of ACD (HR: 1.04, 95% CI: 1.00–1.11) and VaD (HR: 1.09, 95%CI: 1.01–1.18), respectively, in an average of 8.56 ± 2.6 years of follow-up. Second, heterogeneity by gender was observed, and RHR, in both continuous and categorical measures, was widely associated with the greater risks of ACD and VaD in females but not males (Supplementary Materials 5). Third, all the *P* value for interaction between RHR and heart rate reducing medication are > 0.1, and stratified analyses indicated consistently significant between RHR and a greater risk of ACD in subgroups with or without heart rate reducing medication prescription. Interestingly, the only subgroup with heart rate reducing medication prescription presented a significant association between RHR and risk of VaD. However, this could possibly be explained by statistical power discrepancy in the two subgroups, with 25,458 participants in the prescribing group and 311,255 in not prescribing group (Supplementary Materials 5).

### Associations between RHR and cognitive function

Continuous measures of RHR were positively associated with reduced performance on fluid intelligence (OR: 1.015, 95% CI: 1.01–1.02, *P* < 0.001) and reaction time (OR: 1.013, 95% CI: 1.01–1.03, *P* < 0.001). For categorical measures of RHR, the significant associations with worsening cognitive performance were only observed in the test of reaction time, especially in participants with RHR 70–79 bpm (OR: 1.024, 95% CI: 1.01–1.04, *P* < 0.001) and RHR ≥ 80bpm (OR: 1.028, 95% CI:1.01–1.04, *P* < 0.001) (Fig. 1). Similar to the findings of dementia, the associations for continuous measures of RHR and decline in cognitive function remained significant after censoring participants with pre-existing CVDs and with a follow-up duration of less than 5 years. The robustness of the associations was also supported in the subsequent subgroup analysis, despite female- and age-specific models showing insufficient statistical power, potentially due to disproportional representation of outcomes after stratification (Supplementary Materials 6).

### Associations between RHR and brain structure

Since global hippocampal atrophy is considered a common feature of dementia, the linear regression analyses primarily focused on the associations of RHR and hippocampal subfields. Surprisingly, We found that RHR was not related to the volume of the whole hippocampus, but RHR was significantly associated with the decreased volumes of several subfield regions, including fimbria and HATA at both hemispheres; CA3, CA4, GC-ML-DG-head, and hippocampal tail at left hemisphere; and hippocampus fissure in the right hemisphere (Supplementary Material 7).

**Fig. 3:**
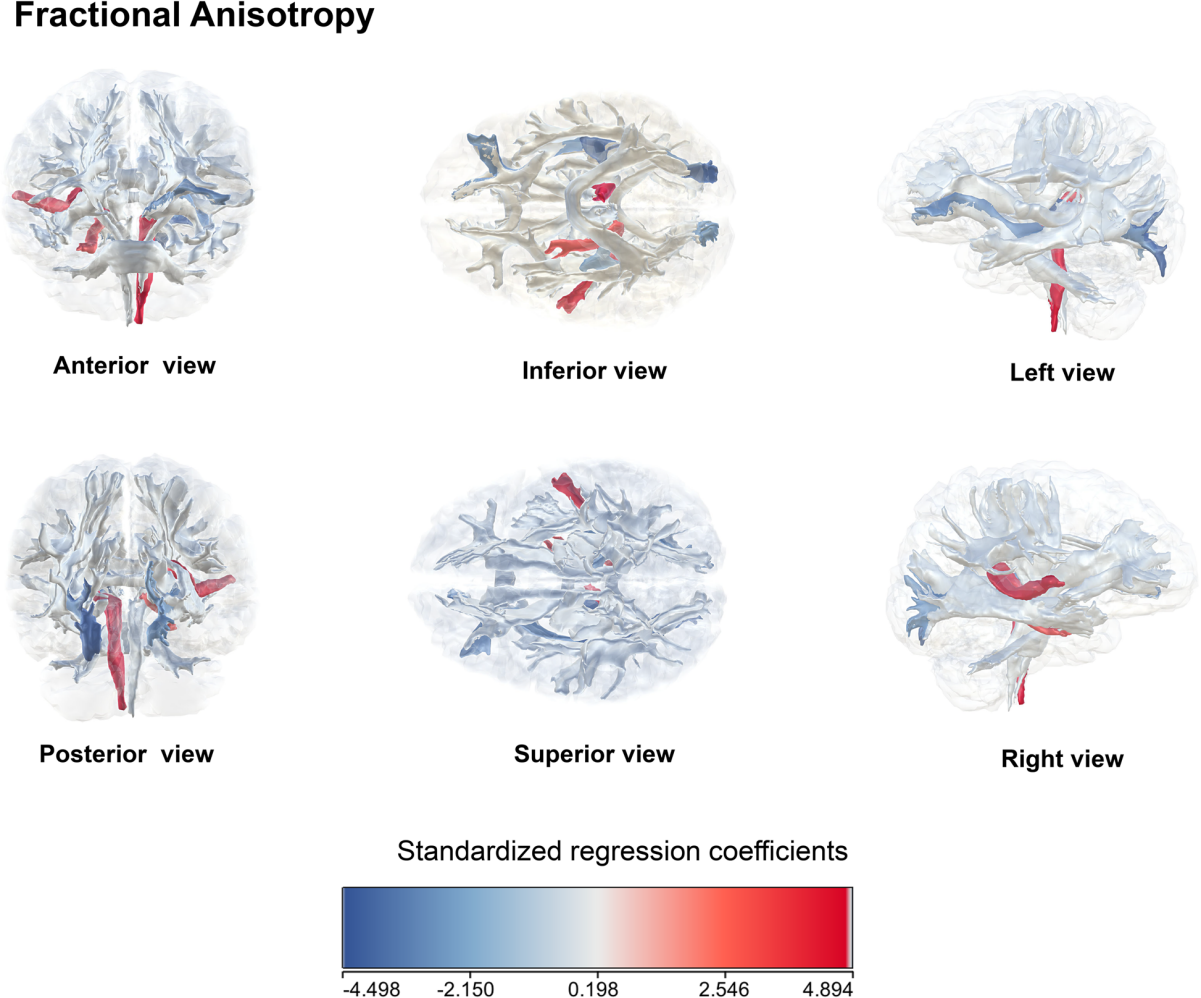
Associations of resting heart rate with white matter integrity indexed by fractional anisotropy measures. Overview of observed standardized regression coefficients for the associations between resting heart rate and fractional anisotropy-based DTI tracts. Standardized regression coefficients reflect the standard deviation (SD) change in FA per standard deviation change resting heart rate. Results were fully adjusted for all covariates

Regarding tract integrity indexed by FA, RHR was associated with left tracts of medial lemniscus radiation, posterior thalamic radiation, inferior frontal-occipital fasciculus radiation and right tracts of acoustic parahippocampal part of cingulum, and posterior thalamic radiation (Fig. 3).

## Discussion

**Fig. 4:**
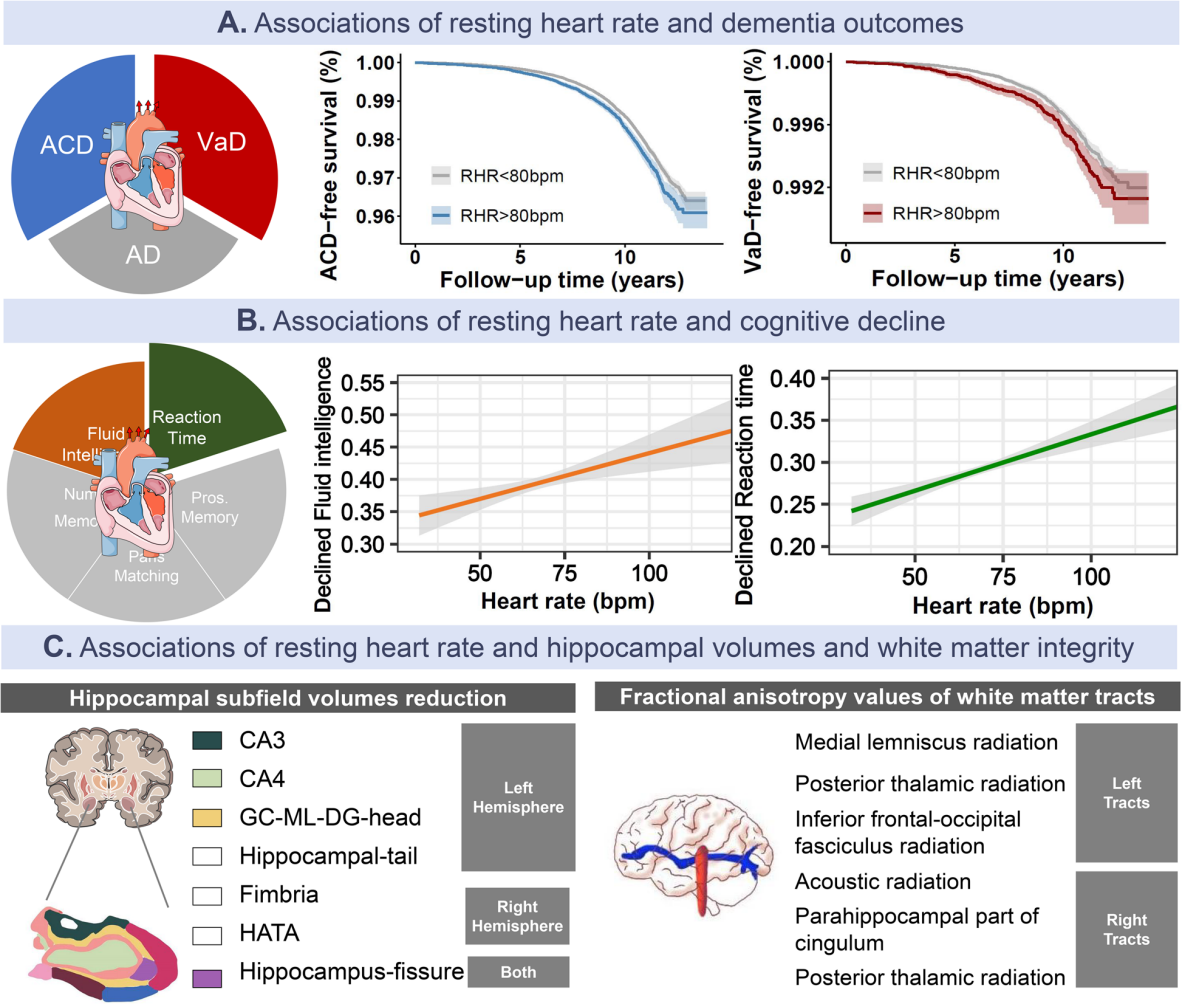
Effects of resting heart rate with dementia, cognitive decline, and brain structures. Content explanation: **A** Kaplan-Meier survival curves for the associations of elevated resting heart rate and risk of dementia. **B** The linear relationships of resting heart rate and cognitive decline in fluid intelligence and reaction time tasks. **C** Associations of resting heart rate with hippocampal subfields and white matter integrity. Left: Colored boxes indicate the corresponding affected regions (certain boxes are presented in white due to the sectional limits). Right: Boxes indicated corresponding affected white matter tracts. Num. Memory, numeric memory tasks; Pros. Memory, prospective memory tasks, GL-ML-DG: granule layer-molecular layer-dentate gyrus boundary, HATA, hippocampal-amygdaloid transition area

By leveraging UK biobank’s advantages of sample size and long-term follow-up, we identified that elevated RHR was associated with an increased risk of ACD and VaD but not AD (Fig. 4). Non-linear analyses indicated J-shape associations for VaD, with RHR being at approximately 70 bpm owning the lowest risk. Under the categorization, participants with RHR ≥ 80 bpm demonstrated greater risks of developing ACD and VaD compared to those with RHR 60–69 bpm. Notably, further subgroup analyses indicated that RHR measures only showed associations with dementia incidents in relatively younger populations (age ≤ 65) and females. Apart from dementia analyses, elevated RHR was linked with worsening performance in fluid intelligence and reaction time, as well as hippocampal atrophy and poor white matter integrity (Fig. 4). Collectively, our findings suggest RHR may be imperative for estimating the risk of dementia and underscore potential connections through structural abnormalities.

As one of the simplest parameters surrogating cardiac autonomic function, RHR has been ubiquitously linked to the increased risks of CVD and all-cause mortality [5, 16, 17]. Although the precise pathophysiological mechanism is still unclear, the evidence, from an epidemiological standpoint, has intuitively facilitated the investigations aiming to unveil the potential entanglement with cognitive dysfunction. The Atherosclerosis Risk in Communities study has described that RHR, measured in mid-life, is prospectively associated with cognitive decline among stroke and atrial fibrillation-free individuals in 20 years of follow-up [6]. Most recently, the Swedish National Study on Aging and Care in Kungsholmen has brought more ascertainment to the findings, where RHR ≥ 80bpm is associated with accelerated cognitive decline and incident dementia after the exclusion of prevalent CVDs [7]. Contrarily, using RHR and heart rate variability to reflect autonomic imbalance, the Framingham study has shown that RHR was only predictive to stroke but not dementia in 10-year follow-up [8]. The Women’s Health Initiative Memory Study, consisting of post-menstrual elderly women (≥ 63 years old), has also failed to establish associations between elevated RHR and cognitive impairment, despite increased brain lesion volumes were indicated on MRI [18]. Nevertheless, our results are mostly coherent with the literature supporting the detrimental effect of RHR on dementia risk.

To depict the implications between RHR and dementia, autonomic dysfunction has been greatly emphasized as a key etiologic factor, primarily due to negative effects on cardiac regulations. Yet, the disparity remains since this symptom is seemingly more apparent across synucleinopathies [19]. However, new evidence has pointed out that autonomic dysfunction may be constantly overlooked, as its occurrence is not unusual in mild cognitive impairment [20], a prodromal phase often leads to the development of dementia [21] as well as other dementia subtypes [22, 23]. The failure of autonomic function is believed to be caused by the cholinergic depletion as part of the dementing process, which RHR, in turn, can be precipitated as a subsequence [24], augmenting the inadequacy of cerebral perfusion on a long-term basis. Moreover, since neurons are exceptionally vulnerable to persistent oxygen deficiency, the brain can undergo acidosis and oxidative stress followed by a cascade of events that aggravates cognitive decline, such as neurovascular dysfunction, white matter abnormalities, and metabolic alterations [21, 25]. Intriguingly, this implication revolving around the cholinergic-autonomic link is mutually supported by the documentation of cholinesterase inhibitors, a commonly administrated drug to improve cognitive function, where the cardioinhibitory side effects can lead to the distortions of heart rates [26]. En masse, we speculate that RHR may be indicative of potential autonomic failure long before the manifestation of cognitive symptoms, and the J-shape relationships observed in our analyses could be partially explained by the cerebral hypoperfusion derived from RHR.

The pathophysiological pathways in relation to CVD can also bring commendable perspectives to associations between RHR and dementia. Abundant data has shown that elevated RHR can impose atrial stiffness and hemodynamic alterations, leading to increased cumulative vascular shear stress, aberrant myocardial mechanical load, and circumferential tensile stress [27–29]. These alterations may progressively facilitate inimical vascular and myocardial remodeling and therefore promotes endothelial dysfunction, acceleration of atherogenesis, and the likelihood of developing conditions susceptible to a higher risk of dementia, such as heart failure and ischemic heart diseases [30]. Increased blood viscosity, platelet activation, and pro-coagulant state prompted by hemodynamic disturbances may also exacerbate the instability of plaques, confronting a greater burden of thromboembolic events [31]. From this point of view, the prominent relations to VaD have implied, to a certain extent, that the underlying mechanism of RHR with dementia is still inclining towards the vascular origin, although whether the associations are eminently mediated by the insidious CVDs cannot be fully precluded.

First, flow velocity (CBFV) and females had higher cerebral blood flow velocity. Moreover, previous research has identified the female sex is an important risk factor. Second, therefore, the results of the stratified analyses toward younger and older age groups also presented few heterogeneities.

A recent review has highlighted the direct and indirect role of amyloid-beta, a typical hallmark of AD, in the acceleration of arterial aging, atherosclerosis at the various stages through activation of inflammatory pathways, which may take place long before the establishment of clinically overt CVDs [25]. To some degree, these findings have invoked that the association between RHR and AD cannot be simply described by a single-directional relationship but rather a reciprocal interaction of systematic mechanisms. As studies have shown that dementia with “pure” characteristics constitutes a rare end upon neurohistological examination, the co-occurrence of vascular and AD pathological processes can act in parallel damages brain tissue, which can easily lead to the misclassification of dementia in accordance with ICD standards. Our results suggest that the RHR may not be conceived as an independent driver of AD. It is possible the null findings are due to the irrelevance to pathological hallmarks or the potential misclassification. However, the significant associations between RHR and ACD, alongside previous literature, have still indicated that RHR is involved, or perhaps affected, in the deteriorating process of AD. Anyhow, we are aware of the fact it is still speculative in nature and demands further evidence.

In the brain imaging analysis pertinent to the hippocampus, although no significant association with the whole hippocampus was observed, we have shown that RHR is associated with decreased hippocampal subfield volumes, implicating that RHR may be a contributory factor to dementia via affecting the hippocampus. Yet, we have failed to establish the relationship between RHR and AD, despite global hippocampal atrophy is considered a major feature. Interestingly, a degree of variance regarding the patterns of atrophy was identified in hippocampal subfields across different dementia types. For instance, asymmetric atrophy in terms of hemispheric and anterior-posterior has been marked between patients with semantic dementia and AD [32]. Disproportional volume alterations in the CA1/2, GC-DG [33], and HATA subregions [34] have also been identified in patients with post-stroke dementia compared to AD, suggesting anoxic-ischemic insults may present distinctive volume reduction on hippocampal subfields. Together, these observations could partly explain the worsening performance in fluid intelligence tasks but relative preservation in other cognitive functions, and the specific clusters of alterations of hippocampal subfields, opposed to widespread volume reduction, among individuals with elevated RHR.

Of note, our subgroup analyses have indicated disparity regarding RHR associations with incident dementia, in which only the female and aged < 65 years groups have yielded robust associations. There are several mechanisms that might explain the findings. First, the differences in neural regulation of resting autonomic function could contribute to the distinctive associations between genders. Previous studies have shown that females tend to have greater parasympathetic modulation of cardiovascular activity compared to males, possibly due to the effects of estrogen [35]. In neuroimaging studies, a higher frequency of heart rate variability, a cardiac vagal activity measure, has been related to brain function. One has demonstrated associations with less perfusion in regions of the medial temporal lobe only in females, particularly in the left parahippocampal gyrus, right hippocampus, and left amygdala [36]. Another has identified correlations with higher cerebral blood velocity in females [36], indicating that females are imposed with greater mechanical stress on the cerebral blood vessels. The findings have suggested that the autonomic control of the female heart is characterized by relatively dominating vagal and parasympathetic activities [35], and, in the case of autonomic dysfunction, females might be more susceptible to greater exposures of cerebral hypoperfusion and cerebrovascular damage than males, resulting in an excess risk of dementia. Second, it is possible that medical treatment is different between genders. A UK biobank study investigating associations between major CVD factors and dementia has observed a dose-response relationship between systolic blood pressure and incident dementia in females only [37], which the authors argued that the findings could be derived from generally lower treatment adherence in females. Third, the only significant estimates observed in the age < 65 groups could be explained by the general downward trend of RHR accompanied by aging. One study monitoring the RHR trajectories in the digital device has shown that average RHR can increase until approximately 50 years of age and then begin to decline, with a narrower magnitude of interindividual variation compared to younger counterparts [38]. It is also worth mentioning that our inclusion of the participants is more representative of healthy individuals, which contradicts similar studies that have a number of older adults carrying more high loads of cardiovascular pathologies and comorbidities [39]. Regardless, the different associations between age might be due to the nature of generally slower heart rate in healthy and older individuals, and an elevated RHR in a younger population could be more reflective of a loss of cardiac reserve, leading to more profound associations with dementia.

Using FA to reflect axonal density and integrity, our findings have suggested that RHR is associated with poor overall white matter health. The posterior thalamic and medial lemniscus radiations associations are in agreement with the prior studies, where the presence of dementia-associated factors may affect these tracts [33]. Notably, a previous DTI study has reported a more widespread white matter damage involving thalamic radiations in VaD compared to AD, which the authors believed the findings may be linked to tracts disconnection and may be beneficial for distinguishing VaD from other dementias (35). With regard to the tracts of the inferior frontal-occipital fasciculus and parahippocampal part of the cingulum, the correlation was typically made in AD [34]. However, a UK biobank study analyzing *APOE* 4 with 22 individual tract-specific FA values has not been able to reach the same conclusion [40]. Although the reason for the discrepancy remains unknown, we suspect it is probably due to underlying mixed pathology, which indirectly argues that at least part of RHR’s contributory pathway to dementia is via vascular routes. For acoustic radiation tracts, one relevant mechanism is the negative compounding effect of vascular pathology on ascending auditory pathway and auditory cortex, which can diminish critical interaction and cognitive processing in the medial temporal lobe and cognitive reserve [41].

This study should be interpreted in the context of several limitations. First, the inclusion criteria of ACD were classified as a collective diagnosis involving several unlisted dementias such as frontal-temporal lobe dementia and Lewy body dementia. We, therefore, could not furtherly distinguish subjects in more precise details. Yet, we were still able to provide the most comprehensive evaluation to date by incorporating AD and VaD, the two most prevalent subtypes, as distinctive outcomes. On top of that, cognitive tasks and imaging findings have allowed us to bring more clarity to the underlying mechanisms. Second, although multiple covariates were considered for adjustment, the effects of residual confounding remain a concern. It was especially challenging in CVDs, as subclinical or undiagnosed cases might contribute to these associations. Regardless, we were still able to minimize the impact by excluding individuals with pre-existing CVDs. Moreover, the regularity/irregularity of RHR caused by some factors (e.g., stimulants, antibiotics, coffee, tea, and fast walking) could be implicated in the indicated associations. It would be more convincing to include Holter data to minimize the confounding effects, which is not provided in this study. Third, RHR was determined single time point at the UKB baseline assessment visit, and restriction of substances, such as coffee and tea, were not strictly limited. Thus, off-clinic measurements of RHR, perhaps using a portable device, might be more suitable for representation. However, the approach has yet to be widely adopted in existing research, considering feasibility and acceptability under the magnitude of cohorts. Fourth, despite the direction of observed effects being very unlikely to diverge, the inaccuracies relating to the measurement of the exposure could still cause minor variations. Fifth, since the imaging data were derived from cross-sectional data, we could not extrapolate the causality and timing linking RHR and structural alterations. There is a possibility that the changes in brain morphometry are affected by other precipitating factors. Still, it has provided complementary support to our longitudinal analysis.

Lastly, the current findings might not be compelling for other ethnic-racial groups because the UK biobank population was mainly constituted with European ancestry. It might be problematic when interpreting the involvement of CVDs since different racial backgrounds could exhibit different prevalence rates. On the other hand, it also minimizes potential bias as the population was strictly controlled.

## Conclusion

In conclusion, RHR ≥ 80bpm was associated with increased risks of ACD and VaD, except AD. A 10-bpm increment of RHR showed a non-linear relationship with VaD, consisting of J-shape associations. Heterogeneities across different sex and age groups were observed, in which RHR measures only showed associations with dementia incidents in relatively younger populations (age ≤ 65) and females. Additionally, RHR was found to be associated with worsening cognitive function, decreased volumes of hippocampus subfields, and poor white matter integrity.

## Supplementary Information

**Additional file 1: Supplementary Materials 1.** Study workflow. **Supplementary Materials 2.** Initial analysis for the associations between resting heart rate and different cognitive tests. **Supplementary Materials 3.** Main analyses. **Supplementary Materials 4.** Testing for non-linearity of resting heart rate effects. **Supplementary Materials 5**. Sensitivity analyses of dementia. **Supplementary Materials 6.** Sensitivity analyses of cognitive decline. **Supplementary Materials 7**. Brain imaging analyses. **Appendix 1**. Field IDs and of UK Biobank. **Appendix 2.** Catalogue of rest heart rate reduction medications in UK biobank.

## Data Availability

According to European law (General Data Protection Regulation), data containing potentially identifying or sensitive patients’ information are restricted. However, for academic researchers, data could be available on request via the UK biobank.

## Abbreviations

ACD: All-cause dementia
AD: Alzheimer’s disease
*APOE* 4: Apolipoprotein E
BMI: Body mass index
CVD: Cardiovascular diseases
DTI: Diffusion tensor imaging
FA: Fractional anisotropy
GL-ML-DG: Granule layer-molecular layer-dentate gyrus boundary
HATA: Hippocampal-amygdaloid transition area
RHR: Resting heart rate
VaD: Vascular dementia
