# Supplementary material for: Associations of resting heart rate with incident dementia, cognition, and brain structure: a prospective cohort study of UK biobank
Source: Alzheimers Res Ther. 2022 Oct 5;14:147. doi: 10.1186/s13195-022-01088-3 (PMC9535982; doi:10.1186/s13195-022-01088-3)
Supplement: Supplementary file 1 — Additional file 1: Supplementary Materials 1. Study workflow. Supplementary Materials 2. Initial analysis for the associations between resting heart rate and different cognitive tests. Supplementary Materials 3. Main analyses. Supplementary Materials 4. Testing for non-linearity of resting heart rate effects. Supplementary Materials 5. Sensitivity analyses of dementia. Supplementary Materials 6. Sensitivity analyses of cognitive decline. Supplementary Materials 7. Brain imaging analyses. Appendix 1. Field IDs and of UK Biobank. Appendix 2. Catalogue of rest heart rate reduction medications in UK biobank. [file 13195_2022_1088_MOESM1_ESM.docx]

**Supplementary Materials – Content**

[1. Supplementary Materials 1- Study workflow 3](#_Toc108690726)

[2. Supplementary Materials 2 – Initial analysis for the associations between resting heart rate and different cognitive tests 4](#_Toc108690727)

[3. Supplementary Materials 3 - Main analyses 5](#_Toc108690728)

[1) Main analysis for the associations between resting heart rate and dementia outcomes 5](#_Toc108690729)

[2) Main analysis for the associations between resting heart rate and cognitive decline 7](#_Toc108690730)

[4. Supplementary Materials 4 – Testing for non-linearity of resting heart rate effects 10](#_Toc108690731)

[1) Non-linearity of resting heart rate in dementia analysis 10](#_Toc108690732)

[2) Non-linearity of resting heart rate in cognitive decline analysis 12](#_Toc108690733)

[3) Non-linearity of resting heart rate in hippocampal subfield volume analysis 13](#_Toc108690734)

[4) Non-linearity of resting heart rate in white matter integrity analysis 15](#_Toc108690735)

[5. Supplementary Materials 5 - Sensitivity analyses of dementia 17](#_Toc108690736)

[1) CVD-restricted models 17](#_Toc108690737)

[2) Age-restricted models 18](#_Toc108690738)

[3) Sex-restricted models 21](#_Toc108690739)

[4) Follow-up-restricted models 24](#_Toc108690740)

[5) Heart rate reducing medication status-restricted models 25](#_Toc108690741)

[6. Supplementary Materials 6 - Sensitivity analyses of cognitive decline 28](#_Toc108690742)

[1) CVD-restricted models 28](#_Toc108690743)

[2) Age-restricted models 29](#_Toc108690744)

[3) Sex-restricted models 31](#_Toc108690745)

[4) Follow-up-restricted models 33](#_Toc108690746)

[7. Supplementary Materials 7 – Brain imaging analyses 34](#_Toc108690747)

[1) The associations between continuous measures of resting heart rate and hippocampal subfield volumes 34](#_Toc108690748)

[2) The associations between categorical measures of resting heart rate and hippocampal subfield volumes 36](#_Toc108690749)

[3) The associations between continuous measures of resting heart rate and fractional anisotropy values of white matter tracts 41](#_Toc108690750)

[4) The associations between categorical measures of resting heart rate and fractional anisotropy values of white matter tracts 43](#_Toc108690751)

[8. *Appendix* 1 – Field IDs and of UK Biobank 47](#_Toc108690752)

[9. *Appendix* 2 – Catalogue of rest heart rate reduction medications in UK biobank 49](#_Toc108690753)

# Supplementary Materials 1- Study workflow


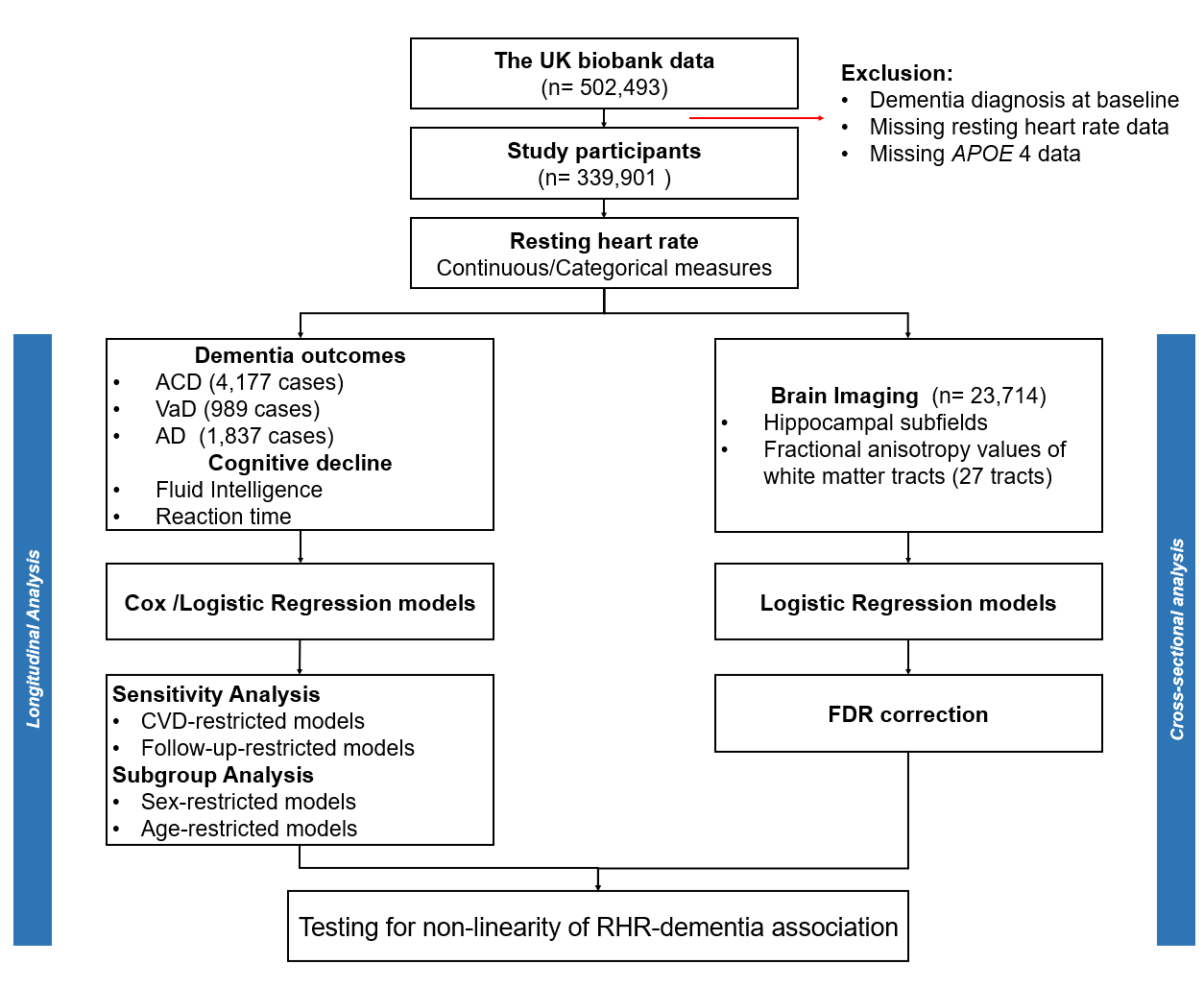


# Supplementary Materials 2 – Initial analysis for the associations between resting heart rate and different cognitive tests

| **SM 2 – Table 1 The associations between resting heart rate and cognitive tests** | | | | | | |
| --- | --- | --- | --- | --- | --- | --- |
| **Cognitive tests** | **Model 1** | | **Model 2** | | **Model 3** | |
|  | **OR (95%CI)** | **FDR - *P*** | **OR (95%CI)** | **FDR - *P*** | **OR (95%CI)** | **FDR - *P*** |
| **Total sample** |  |  |  |  |  |  |
| Numeric memory | 1.05 (0.98-1.12) | 0.302 | 1.01 (0.99-1.03) | 0.702 | 1.014 (1-1.03) | 0.176 |
| Fluid intelligence | 1.059 (1.02-1.1) | 0.003 | 1.014 (1.01-1.02) | 0.003 | 1.015 (1.01-1.02) | 0.001 |
| Prospective memory | 1 (0.94-1.07) | 0.993 | 1 (1-1) | <0.001 | 1.001 (1-1.01) | 0.796 |
| Pairs matching | 1.016 (0.99-1.04) | 0.383 | 1.002 (1-1.01) | 0.269 | 1.002 (1-1.01) | 0.476 |
| Reaction time | 1.062 (1.04-1.09) | <0.001 | 1.013 (1.01-1.02) | <0.001 | 1.013 (1.01-1.02) | <0.001 |
| Cognition | 0.995 (0.93-1.07) | 1.000 | 0.999 (0.98-1.01) | 1.000 | 0.999 (0.98-1.02) | 1.000 |
| Abbreviations: RHR, resting heart rate; HR, hazard ratios, 95CI%, 95% confidential intervals *Model 1: Adjusted for age, sex, education, and *APOE* 4 Status *Model 2: Adjusted for covariates of model 1 + smoking, physical activity, and BMI *Model 3: Adjusted for all covariates | | | | | | |

# Supplementary Materials 3 - Main analyses

## Main analysis for the associations between resting heart rate and dementia outcomes

| **SM 3 - Table 1 The associations between resting heart rate and dementia outcomes** | | | | | | | | | |
| --- | --- | --- | --- | --- | --- | --- | --- | --- | --- |
|  | **RHR** | **No. of participants** | **No. dementia Cases** | **Model 1** | | **Model 2** | | **Model 3** | |
|  |  |  |  | **HR (95%CI)** | ***P*** | **HR (95%CI)** | ***P*** | **HR (95%CI)** | ***P*** |
| ACD | RHR per 10 bpm increment | 339901 | 4177 | 1.06 (1.03-1.08) | <0.001 | 1.05 (1.03-1.08) | <0.001 | 1.06 (1.03-1.09) | <0.001 |
|  | Categorical RHR |  |  |  |  |  |  |  |  |
|  | RHR <60 bpm | 63711 | 784 | 1 (0.92-1.1) | 0.960 | 1.01 (0.92-1.1) | 0.852 | 0.95 (0.87-1.04) | 0.290 |
|  | RHR 60-69 bpm | 118650 | 1341 | Reference |  | Reference |  | Reference |  |
|  | RHR 70-79 bpm | 95571 | 1144 | 1.04 (0.97-1.13) | 0.277 | 1.04 (0.96-1.12) | 0.338 | 1.05 (0.97-1.13) | 0.256 |
|  | RHR >80 bpm | 61969 | 908 | 1.21 (1.11-1.31) | <0.001 | 1.2 (1.1-1.3) | <0.001 | 1.18 (1.08-1.28) | <0.001 |
| AD | RHR per 10 bpm increment | 337561 | 1837 | 1.02 (0.98-1.06) | 0.268 | 1.03 (0.99-1.07) | 0.129 | 1.03 (0.99-1.07) | 0.216 |
|  | Categorical RHR |  |  | 1.03 (0.9-1.17) | 0.703 | 1.02 (0.9-1.17) | 0.721 | 1 (0.88-1.15) | 0.943 |
|  | RHR <60 bpm | 63273 | 346 |  |  |  |  |  |  |
|  | RHR 60-69 bpm | 117899 | 590 | Reference |  | Reference |  | Reference |  |
|  | RHR 70-79 bpm | 94968 | 541 | 1.11 (0.99-1.25) | 0.085 | 1.12 (1-1.26) | 0.060 | 1.11 (0.99-1.25) | 0.070 |
|  | RHR >80 bpm | 61421 | 360 | 1.08 (0.95-1.23) | 0.251 | 1.11 (0.97-1.27) | 0.124 | 1.08 (0.94-1.23) | 0.263 |
| VaD | RHR per 10 bpm increment | 336713 | 989 | 1.07 (1.01-1.12) | 0.012 | 1.04 (0.99-1.1) | 0.115 | 1.07 (1.01-1.12) | 0.017 |
|  | Categorical RHR |  |  |  |  |  |  |  |  |
|  | RHR <60 bpm | 63146 | 219 | 1.23 (1.03-1.47) | 0.020 | 1.25 (1.05-1.5) | 0.012 | 1.11 (0.93-1.33) | 0.256 |
|  | RHR 60-69 bpm | 117601 | 292 | Reference |  | Reference |  | Reference |  |
|  | RHR 70-79 bpm | 94676 | 249 | 1.06 (0.89-1.25) | 0.522 | 1.03 (0.87-1.22) | 0.751 | 1.05 (0.88-1.24) | 0.594 |
|  | RHR >80 bpm | 61290 | 229 | 1.41 (1.19-1.68) | <0.001 | 1.32 (1.11-1.57) | 0.002 | 1.29 (1.08-1.54) | 0.005 |
| Abbreviations: RHR, resting heart rate; ACD, all-cause dementia; AD, Alzheimer's disease; VaD, vascular dementia; HR, hazard ratios, 95CI%, 95% confidential intervals *Model 1: Adjusted for age, sex, education, and *APOE* 4 Status *Model 2: Adjusted for covariates of model 1 + smoking, physical activity, and BMI *Model 3: Adjusted for all covariates | | | | | | | | | |

| **SM 3 - Table 2 The associations between resting heart rate and dementia outcomes before exclusion of participants without *APOE* genotype information** | | | | | | | | | |
| --- | --- | --- | --- | --- | --- | --- | --- | --- | --- |
|  | **RHR** | **No. of participants** | **No. dementia Cases** | **Model 1** | | **Model 2** | | **Model 3** | |
|  |  |  |  | **HR (95%CI)** | ***P*** | **HR (95%CI)** | ***P*** | **HR (95%CI)** | ***P*** |
| ACD | RHR per 10 bpm increment | 393289 | 4795 | 1.05 (1.03-1.07) | <0.001 | 1.05 (1.02-1.07) | <0.001 | 1.06 (1.04-1.09) | <0.001 |
|  | Categorical RHR |  |  |  |  |  |  |  |  |
|  | RHR <60 bpm | 72780 | 889 | 1 (0.92-1.09) | 0.980 | 1.01 (0.93-1.09) | 0.882 | 0.95 (0.88-1.04) | 0.278 |
|  | RHR 60-69 bpm | 137346 | 1560 | Reference |  | Reference |  | Reference |  |
|  | RHR 70-79 bpm | 111135 | 1296 | 1.01 (0.94-1.08) | 0.840 | 1 (0.93-1.08) | 0.925 | 1.01 (0.94-1.09) | 0.806 |
|  | RHR >80 bpm | 72028 | 1050 | 1.18 (1.09-1.28) | <0.001 | 1.17 (1.08-1.27) | <0.001 | 1.15 (1.06-1.24) | 0.001 |
| AD | RHR per 10 bpm increment | 390582 | 2088 | 1 (0.96-1.03) | 0.840 | 1.01 (0.97-1.04) | 0.786 | 1.01 (0.97-1.04) | 0.775 |
|  | Categorical RHR |  |  |  |  |  |  |  |  |
|  | RHR <60 bpm | 72292 | 401 | 1.06 (0.94-1.2) | 0.355 | 1.06 (0.94-1.2) | 0.369 | 1.04 (0.91-1.18) | 0.582 |
|  | RHR 60-69 bpm | 136468 | 682 | Reference |  | Reference |  | Reference |  |
|  | RHR 70-79 bpm | 110441 | 602 | 1.06 (0.95-1.18) | 0.314 | 1.07 (0.96-1.19) | 0.238 | 1.07 (0.95-1.19) | 0.255 |
|  | RHR >80 bpm | 71381 | 403 | 1.03 (0.91-1.16) | 0.657 | 1.06 (0.93-1.2) | 0.374 | 1.03 (0.91-1.17) | 0.653 |
| VaD | RHR per 10 bpm increment | 389628 | 1134 | 1.06 (1.02-1.12) | 0.010 | 1.04 (0.99-1.09) | 0.088 | 1.07 (1.02-1.12) | 0.006 |
|  | Categorical RHR |  |  |  |  |  |  |  |  |
|  | RHR <60 bpm | 72125 | 234 | 1.13 (0.96-1.34) | 0.139 | 1.15 (0.97-1.36) | 0.099 | 1.03 (0.87-1.22) | 0.727 |
|  | RHR 60-69 bpm | 136135 | 349 | Reference |  | Reference |  | Reference |  |
|  | RHR 70-79 bpm | 110128 | 289 | 1.02 (0.87-1.19) | 0.837 | 0.99 (0.85-1.16) | 0.932 | 1.01 (0.86-1.18) | 0.920 |
|  | RHR >80 bpm | 71240 | 262 | 1.33 (1.13-1.56) | 0.001 | 1.25 (1.07-1.47) | 0.006 | 1.21 (1.03-1.42) | 0.024 |
| Abbreviations: RHR, resting heart rate; ACD, all-cause dementia; AD, Alzheimer's disease; VaD, vascular dementia; HR, hazard ratios, 95CI%, 95% confidential intervals *Model 1: Adjusted for age, sex, and education *Model 2: Adjusted for covariates of model 1 + smoking, physical activity, and BMI *Model 3: Adjusted for all covariates | | | | | | | | | |

## Main analysis for the associations between resting heart rate and cognitive decline

| **SM 3 – Table 2 The associations between resting heart rate and cognitive decline** | | | | | | | |
| --- | --- | --- | --- | --- | --- | --- | --- |
|  | **RHR** | **Model 1** | | **Model 2** | | **Model 3** | |
|  |  | **OR (95%CI)** | **FDR - *P*** | **OR (95%CI)** | **FDR - *P*** | **OR (95%CI)** | **FDR - *P*** |
| **Fluid intelligence** | RHR per 10 bpm increment | 1.059 (1.02-1.1) | 0.001 | 1.014 (1.01-1.02) | 0.002 | 1.015 (1.01-1.02) | 0.001 |
|  | Categorical RHR |  |  |  |  |  |  |
|  | RHR <60 bpm | 0.893 (0.81-0.99) | 0.054 | 0.974 (0.95-1) | 0.056 | 0.972 (0.95-1) | 0.040 |
|  | RHR 60-69 bpm | Reference |  | Reference |  | Reference |  |
|  | RHR 70-79 bpm | 0.96 (0.87-1.06) | 0.418 | 0.99 (0.97-1.01) | 0.410 | 0.991 (0.97-1.01) | 0.459 |
|  | RHR >80 bpm | 1.115 (0.99-1.26) | 0.082 | 1.026 (1-1.06) | 0.089 | 1.027 (1-1.06) | 0.080 |
|  | RHR per 10 bpm increment | 1.062 (1.04-1.09) | <0.001 | 1.013 (1.01-1.02) | <0.001 | 1.013 (1.01-1.02) | <0.001 |
|  | Categorical RHR |  |  |  |  |  |  |
|  | RHR <60 bpm | 0.948 (0.89-1.01) | 0.123 | 0.989 (0.98-1) | 0.115 | 0.988 (0.97-1) | 0.078 |
| **Reaction time** | RHR 60-69 bpm | Reference |  | Reference |  | Reference |  |
|  | RHR 70-79 bpm | 1.12 (1.05-1.19) | <0.001 | 1.024 (1.01-1.04) | <0.001 | 1.024 (1.01-1.04) | <0.001 |
|  | RHR >80 bpm | 1.145 (1.06-1.24) | <0.001 | 1.028 (1.01-1.04) | 0.002 | 1.028 (1.01-1.04) | 0.002 |
| Abbreviations: RHR, resting heart rate; HR, hazard ratios, 95CI%, 95% confidential intervals *Model 1: Adjusted for age, sex, education, and *APOE* 4 Status *Model 2: Adjusted for covariates of model 1 + smoking, physical activity, and BMI *Model 3: Adjusted for all covariates | | | | | | | |

| **SM 3 – Table 3 The associations between resting heart rate and cognitive decline before exclusion of participants without *APOE* genotype information** | | | | | | | |
| --- | --- | --- | --- | --- | --- | --- | --- |
|  | **RHR** | **Model 1** | | **Model 2** | | **Model 3** | |
|  |  | **OR (95%CI)** | **FDR - *P*** | **OR (95%CI)** | **FDR - *P*** | **OR (95%CI)** | **FDR - *P*** |
| **Fluid intelligence** | RHR per 10 bpm increment | 1.006 (1-1.01) | <0.001 | 1.006 (1-1.01) | 0.002 | 1.006 (1-1.01) | <0.001 |
|  | Categorical RHR |  |  |  |  |  |  |
|  | RHR <60 bpm | 1.104 (1.01-1.21) | 0.078 | 1.102 (1-1.21) | 0.045 | 1.11 (1.01-1.22) | 0.031 |
|  | RHR 60-69 bpm | Reference |  | Reference |  | Reference |  |
|  | RHR 70-79 bpm | 1.07 (0.97-1.19) | 0.199 | 1.064 (0.96-1.18) | 0.242 | 1.078 (0.97-1.2) | 0.162 |
|  | RHR >80 bpm | 1.25 (1.1-1.42) | <0.001 | 1.24 (1.09-1.41) | 0.001 | 1.259 (1.11-1.43) | <0.001 |
|  | RHR per 10 bpm increment | 1.006 (1-1.01) | <0.001 | 1.006 (1-1.01) | <0.001 | 1.006 (1-1.01) | <0.001 |
|  | Categorical RHR |  |  |  |  |  |  |
|  | RHR <60 bpm | 1.069 (1-1.14) | 0.042 | 1.072 (1.01-1.14) | 0.066 | 1.079 (1.01-1.15) | 0.042 |
| **Reaction time** | RHR 60-69 bpm | Reference |  | Reference |  | Reference |  |
|  | RHR 70-79 bpm | 1.157 (1.08-1.24) | <0.001 | 1.16 (1.08-1.24) | <0.001 | 1.17 (1.09-1.25) | <0.001 |
|  | RHR >80 bpm | 1.204 (1.11-1.3) | <0.001 | 1.206 (1.11-1.31) | <0.001 | 1.217 (1.12-1.32) | <0.001 |
| Abbreviations: RHR, resting heart rate; HR, hazard ratios, 95CI%, 95% confidential intervals *Model 1: Adjusted for age, sex and education *Model 2: Adjusted for covariates of model 1 + smoking, physical activity, and BMI *Model 3: Adjusted for all covariates | | | | | | | |

| **SM 3 – Table 4 The associations between resting heart rate and change in cognitive scores** | | | | | | | |
| --- | --- | --- | --- | --- | --- | --- | --- |
|  | **RHR** | **Model 1** | | **Model 2** | | **Model 3** | |
|  |  | **OR (95%CI)** | **FDR - *P*** | **OR (95%CI)** | **FDR - *P*** | **OR (95%CI)** | **FDR - *P*** |
| **Fluid intelligence** | RHR per 10 bpm increment | 0.961 (0.93-0.99) | 0.009 | 0.963 (0.93-0.99) | 0.015 | 0.958 (0.93-0.99) | 0.006 |
|  | Categorical RHR |  |  |  |  |  |  |
|  | RHR <60 bpm | 1.063 (0.98-1.16) | 0.157 | 1.061 (0.97-1.16) | 0.170 | 1.07 (0.98-1.17) | 0.120 |
|  | RHR 60-69 bpm | Reference |  | Reference |  | Reference |  |
|  | RHR 70-79 bpm | 1.025 (0.94-1.11) | 0.558 | 1.027 (0.94-1.12) | 0.528 | 1.023 (0.94-1.11) | 0.591 |
|  | RHR >80 bpm | 0.905 (0.82-1.01) | 0.063 | 0.909 (0.82-1.01) | 0.076 | 0.902 (0.81-1) | 0.057 |
|  | RHR per 10 bpm increment | 0.053 (0.02-0.15) | <0.001 | 0.061 (0.02-0.18) | <0.001 | 0.052 (0.02-0.16) | <0.001 |
|  | Categorical RHR |  |  |  |  |  |  |
|  | RHR <60 bpm | 11.801 (0.49-284.01) | 0.256 | 11.389 (0.47-276.68) | 0.270 | 14.993 (0.6-372.43) | 0.198 |
| **Reaction time** | RHR 60-69 bpm | Reference |  | Reference |  | Reference |  |
|  | RHR 70-79 bpm | 0.007 (0-0.14) | 0.001 | 0.008 (0-0.17) | 0.004 | 0.007 (0-0.15) | 0.002 |
|  | RHR >80 bpm | 0.001 (0-0.02) | <0.001 | 0.001 (0-0.04) | <0.001 | 0.001 (0-0.03) | <0.001 |
| Abbreviations: RHR, resting heart rate; HR, hazard ratios, 95CI%, 95% confidential intervals *Model 1: Adjusted for age, sex, education, and *APOE* 4 Status *Model 2: Adjusted for covariates of model 1 + smoking, physical activity, and BMI *Model 3: Adjusted for all covariates | | | | | | | |

# Supplementary Materials 4 – Testing for non-linearity of resting heart rate effects

## Non-linearity of resting heart rate in dementia analysis

| **SM 4 – 1 Table Testing for non-linearity of resting heart rate effects in dementia analysis** | | | | |
| --- | --- | --- | --- | --- |
|  |  | **Model 1** | **Model 2** | **Model 3** |
|  |  | ***P*** | ***P*** | ***P*** |
| ACD | Main cox regression models | 0.015 | 0.010 | 0.335 |
|  | CVD-restricted models | 0.050 | 0.039 | 0.225 |
|  | Age-restricted models |  |  |  |
|  | Age <65 | 0.220 | 0.190 | 0.985 |
|  | Age >65 | 0.026 | 0.021 | 0.193 |
|  | Sex-restricted models |  |  |  |
|  | Male | 0.025 | 0.024 | 0.319 |
|  | Female | 0.078 | 0.066 | 0.527 |
|  | Follow-up restricted models | 0.224 | 0.190 | 0.868 |
| AD | Main cox regression models | 0.875 | 0.285 | 0.929 |
|  | CVD-restricted models | 0.840 | 0.729 | 0.834 |
|  | Age-restricted models |  |  |  |
|  | Age <65 | 0.262 | 0.297 | 0.165 |
|  | Age >65 | 0.152 | 0.120 | 0.233 |
|  | Sex-restricted models |  |  |  |
|  | Male | 0.988 | 0.922 | 0.667 |
|  | Female | 0.705 | 0.672 | 0.911 |
|  | Follow-up restricted models | 0.684 | 0.588 | 0.908 |
| VaD | Main cox regression models | <0.001 | <0.001 | 0.001 |
|  | CVD-restricted models | <0.001 | <0.001 | 0.003 |
|  | Age-restricted models |  |  |  |
|  | Age <65 | <0.001 | <0.001 | 0.018 |
|  | Age >65 | 0.020 | 0.024 | 0.191 |
|  | Sex-restricted models |  |  |  |
|  | Male | <0.001 | <0.001 | 0.001 |
|  | Female | 0.009 | 0.009 | 0.219 |
|  | Follow-up restricted models | 0.003 | 0.003 | 0.003 |
| Abbreviations: RHR, resting heart rate; ACD, all-cause dementia; AD, Alzheimer's disease; VaD, vascular dementia; HR, hazard ratios, 95CI%, 95% confidential intervals *Model 1: Adjusted for age, sex, education, and *APOE* 4 Status *Model 2: Adjusted for covariates of model 1 + smoking, physical activity, and BMI *Model 3: Adjusted for all covariates | | | | |

## Non-linearity of resting heart rate in cognitive decline analysis

| **SM 4 - Table 2 Testing for non-linearity of resting heart rate effects in cognitive decline analysis** | | | | |
| --- | --- | --- | --- | --- |
|  |  | **Model 1** | **Model 2** | **Model 3** |
|  |  | **FDR - *P*** | **FDR - *P*** | **FDR - *P*** |
| Fluid intelligence | Main cox regression models | 0.760 | 0.940 | 0.823 |
|  | Baseline CVD-restricted models | 0.976 | 0.663 | 0.604 |
|  | Age-restricted models |  |  |  |
|  | Age <65 | 1.000 | 0.892 | 0.999 |
|  | Age >65 | 1.000 | 0.499 | 0.902 |
|  | Sex-restricted models |  |  |  |
|  | Male | 0.980 | 0.732 | 0.587 |
|  | Female | 0.783 | 0.915 | 0.927 |
|  | Follow-up restricted models | 0.617 | 0.909 | 0.998 |
| Reaction time | Main cox regression models | 1.000 | 0.550 | 0.386 |
|  | Baseline CVD-restricted models | 1.000 | 0.680 | 0.686 |
|  | Age-restricted models |  |  |  |
|  | Age <65 | 0.654 | 0.628 | 0.432 |
|  | Age >65 | 0.868 | 1.000 | 0.792 |
|  | Sex-restricted models |  |  |  |
|  | Male | 1.000 | 1.000 | 0.928 |
|  | Female | 1.000 | 0.828 | 0.626 |
|  | Follow-up restricted models | 1.000 | 0.486 | 0.306 |
| Abbreviations: RHR, resting heart rate; HR, hazard ratios, 95CI%, 95% confidential intervals *Model 1: Adjusted for age, sex, education, and *APOE* 4 Status *Model 2: Adjusted for covariates of model 1 + smoking, physical activity, and BMI *Model 3: Adjusted for all covariates | | | | |

## Non-linearity of resting heart rate in hippocampal subfield volume analysis

| **SM 4 - Table 3 Testing for non-linearity of resting heart rate effects in hippocampal subfield volume analysis** | |
| --- | --- |
| **Hippocampal volume** | ***P*** |
| **Left hemisphere** |  |
| Paralaminar-nucleus | 0.013 |
| Hippocampal-tail | 0.036 |
| subiculum-body | 0.469 |
| CA1-body | 0.475 |
| subiculum-head | 0.202 |
| hippocampal-fissure | 0.564 |
| presubiculum-head | 0.025 |
| CA1-head | 0.010 |
| presubiculum-body | 0.653 |
| GC-ML-DG-head | 0.029 |
| CA3-body | 0.153 |
| GC-ML-DG-body | 0.255 |
| CA4-head | 0.038 |
| CA4-body | 0.398 |
| fimbria | 0.309 |
| CA3-head | 0.051 |
| HATA | 0.065 |
| Whole-hippocampal-body | 0.291 |
| Whole-hippocampal-head | 0.020 |
| Whole-hippocampus | 0.026 |
| **Right hemisphere** |  |
| Paralaminar-nucleus | 0.028 |
| Hippocampal-tail | 0.068 |
| subiculum-body | 0.175 |
| CA1-body | 0.017 |
| subiculum-head | 0.377 |
| hippocampal-fissure | 0.288 |
| presubiculum-head | 0.329 |
| CA1-head | 0.069 |
| presubiculum-body | 0.156 |
| GC-ML-DG-head | 0.077 |
| CA3-body | 0.380 |
| GC-ML-DG-body | 0.583 |
| CA4-head | 0.122 |
| CA4-body | 0.583 |
| fimbria | 0.499 |
| CA3-head | 0.270 |
| HATA | 0.483 |
| Whole-hippocampal-body | 0.077 |
| Whole-hippocampal-head | 0.145 |
| Whole-hippocampus | 0.044 |

## Non-linearity of resting heart rate in white matter integrity analysis

| **SM 4- Table 4 Testing for non-linearity of resting heart rate effects in white matter integrity analysis** | |
| --- | --- |
| **White matter microstructure** | ***P*** |
| **Left tract** |  |
| Acoustic radiation | 0.951 |
| Anterior thalamic radiation | 0.402 |
| Cingulate gyrus part of cingulum | 0.709 |
| Parahippocampal part of cingulum | 0.310 |
| Corticospinal tract | 0.215 |
| Inferior fronto-occipital fasciculus | 0.169 |
| Inferior longitudinal fasciculus | 0.149 |
| Medial lemniscus | 0.486 |
| Posterior thalamic radiation | 0.495 |
| Superior longitudinal fasciculus | 0.748 |
| Superior thalamic radiation | 0.022 |
| Uncinate fasciculus | 0.422 |
| **Right tract** |  |
| Acoustic radiation | 0.111 |
| Anterior thalamic radiation | 0.135 |
| Cingulate gyrus part of cingulum | 0.520 |
| Parahippocampal part of cingulum | 0.484 |
| Corticospinal tract | 0.115 |
| Inferior fronto-occipital fasciculus | 0.108 |
| Inferior longitudinal fasciculus | 0.436 |
| Medial lemniscus | 0.720 |
| Posterior thalamic radiation | 0.750 |
| Superior longitudinal fasciculus | 0.956 |
| Superior thalamic radiation | 0.007 |
| Uncinate fasciculus | 0.023 |
| **Other tract** |  |
| Forceps major | 0.988 |
| Forceps minor | 0.243 |
| Middle cerebellar peduncle | 0.605 |

# Supplementary Materials 5 - Sensitivity analyses of dementia

## CVD-restricted models

| **SM 5 - Table 1 The associations between resting heart rate and dementia outcomes in CVD-restricted models** | | | | | | | | | |
| --- | --- | --- | --- | --- | --- | --- | --- | --- | --- |
|  | **RHR** | **No. of participants** | **No. dementia cases** | **Model 1** | | **Model 2** | | **Model 3** | |
|  |  |  |  | **HR (95%CI)** | ***P*** | **HR (95%CI)** | ***P*** | **HR (95%CI)** | ***P*** |
| ACD | RHR per 10 bpm increment | 317510 | 3404 | 1.05 (1.02-1.08) | 0.001 | 1.05 (1.02-1.08) | 0.001 | 1.05 (1.02-1.08) | 0.001 |
|  | Categorical RHR |  |  | 0.97 (0.88-1.08) | 0.614 | 0.98 (0.89-1.08) | 0.679 | 0.95 (0.86-1.05) | 0.306 |
|  | RHR <60 bpm | 57164 | 575 |  |  |  |  |  |  |
|  | RHR 60-69 bpm | 111951 | 1131 | Reference |  | Reference |  | Reference |  |
|  | RHR 70-79 bpm | 90616 | 966 | 1.03 (0.94-1.12) | 0.529 | 1.03 (0.94-1.12) | 0.547 | 1.04 (0.95-1.13) | 0.431 |
|  | RHR >80 bpm | 57779 | 732 | 1.15 (1.05-1.27) | 0.003 | 1.15 (1.05-1.27) | 0.003 | 1.15 (1.04-1.26) | 0.005 |
| AD | RHR per 10 bpm increment | 315672 | 1566 | 1.02 (0.98-1.07) | 0.290 | 1.04 (0.99-1.08) | 0.108 | 1.03 (0.98-1.07) | 0.238 |
|  | Categorical RHR |  |  |  |  |  |  |  |  |
|  | RHR <60 bpm | 56851 | 262 | 0.98 (0.84-1.13) | 0.754 | 0.97 (0.84-1.13) | 0.697 | 0.97 (0.84-1.13) | 0.720 |
|  | RHR 60-69 bpm | 111342 | 522 | Reference |  | Reference |  | Reference |  |
|  | RHR 70-79 bpm | 90117 | 467 | 1.07 (0.94-1.21) | 0.306 | 1.08 (0.96-1.23) | 0.215 | 1.08 (0.95-1.22) | 0.246 |
|  | RHR >80 bpm | 57362 | 315 | 1.07 (0.93-1.23) | 0.348 | 1.11 (0.96-1.28) | 0.154 | 1.08 (0.94-1.25) | 0.276 |
| VaD | RHR per 10 bpm increment | 314807 | 701 | 1.92 (1.04-3.54) | 0.037 | 1.56 (0.84-2.89) | 0.163 | 1.91 (1.01-3.62) | 0.047 |
|  | Categorical RHR |  |  |  |  |  |  |  |  |
|  | RHR <60 bpm | 56729 | 140 | 1.24 (1-1.54) | 0.048 | 1.26 (1.02-1.57) | 0.032 | 1.17 (0.94-1.45) | 0.168 |
|  | RHR 60-69 bpm | 111031 | 211 | Reference |  | Reference |  | Reference |  |
|  | RHR 70-79 bpm | 89836 | 186 | 1.06 (0.87-1.3) | 0.541 | 1.04 (0.85-1.27) | 0.700 | 1.07 (0.87-1.3) | 0.525 |
|  | RHR >80 bpm | 57211 | 164 | 1.39 (1.13-1.7) | 0.002 | 1.31 (1.07-1.61) | 0.010 | 1.31 (1.06-1.61) | 0.012 |
| Abbreviations: RHR, resting heart rate; ACD, all-cause dementia; AD, Alzheimer's disease; VaD, vascular dementia; HR, hazard ratios, 95CI%, 95% confidential intervals *Model 1: Adjusted for age, sex, education, and *APOE* 4 Status *Model 2: Adjusted for covariates of model 1 + smoking, physical activity, and BMI *Model 3: Adjusted for all covariates | | | | | | | | | |

## Age-restricted models

| **SM 5 – Table 2a Interaction terms for age** | | | |
| --- | --- | --- | --- |
| **P for interaction** | **Model 1** | **Model 2** | **Model 3** |
| ACD | 0.2575 | 0.2643 | 0.4297 |
| AD | 0.4370 | 0.3685 | 0.4345 |
| VD | 0.2020 | 0.2573 | 0.4292 |
| Abbreviations: ACD, all-cause dementia; AD, Alzheimer's disease; VaD, vascular dementia *Model 1: Adjusted for age, sex, education, and *APOE* 4 Status *Model 2: Adjusted for covariates of model 1 + smoking, physical activity, and BMI *Model 3: Adjusted for all covariates | | | |

| **SM 5 - Table 2a The associations between resting heart rate and dementia outcomes in age restricted models (Age≥65)** | | | | | | | | | |
| --- | --- | --- | --- | --- | --- | --- | --- | --- | --- |
|  | **RHR** | **No. of participants** | **No. dementia cases** | **Model 1** | | **Model 2** | | **Model 3** | |
|  |  |  |  | **HR (95%CI)** | ***P*** | **HR (95%CI)** | ***P*** | **HR (95%CI)** | ***P*** |
|  | RHR per 10 bpm increment | 48166 | 1665 | 1.03 (0.99-1.07) | 0.182 | 1.03 (0.99-1.07) | 0.183 | 1.04 (1-1.08) | 0.068 |
|  | Categorical RHR |  |  |  |  |  |  |  |  |
| ACD | RHR <60 bpm | 8340 | 308 | 1.11 (0.96-1.28) | 0.152 | 1.11 (0.97-1.28) | 0.142 | 1.07 (0.92-1.23) | 0.374 |
|  | RHR 60-69 bpm | 16371 | 528 | Reference |  | Reference |  | Reference |  |
|  | RHR 70-79 bpm | 13803 | 466 | 1.06 (0.94-1.2) | 0.334 | 1.07 (0.94-1.21) | 0.291 | 1.08 (0.95-1.22) | 0.232 |
|  | RHR >80 bpm | 9652 | 363 | 1.2 (1.05-1.37) | 0.009 | 1.2 (1.05-1.37) | 0.008 | 1.2 (1.05-1.38) | 0.007 |
|  | RHR per 10 bpm increment | 54267 | 940 | 1 (0.95-1.06) | 0.868 | 1.01 (0.96-1.07) | 0.702 | 1.01 (0.95-1.07) | 0.757 |
|  | Categorical RHR |  |  |  |  |  |  |  |  |
| AD | RHR <60 bpm | 10360 | 203 | 1.2 (1.01-1.44) | 0.044 | 1.21 (1.01-1.45) | 0.042 | 1.18 (0.98-1.42) | 0.076 |
|  | RHR 60-69 bpm | 18203 | 286 | Reference |  | Reference |  | Reference |  |
|  | RHR 70-79 bpm | 15039 | 257 | 1.08 (0.92-1.28) | 0.350 | 1.1 (0.93-1.3) | 0.285 | 1.09 (0.92-1.29) | 0.331 |
|  | RHR >80 bpm | 10665 | 194 | 1.18 (0.98-1.41) | 0.079 | 1.2 (1-1.45) | 0.048 | 1.17 (0.98-1.41) | 0.090 |
|  | RHR per 10 bpm increment | 53840 | 513 | 1.03 (0.96-1.1) | 0.435 | 1.01 (0.94-1.09) | 0.731 | 1.03 (0.96-1.11) | 0.358 |
|  | Categorical RHR |  |  |  |  |  |  |  |  |
| VaD | RHR <60 bpm | 10276 | 119 | 1.25 (0.98-1.59) | 0.068 | 1.27 (0.99-1.61) | 0.056 | 1.14 (0.89-1.46) | 0.299 |
|  | RHR 60-69 bpm | 18069 | 152 | Reference |  | Reference |  | Reference |  |
|  | RHR 70-79 bpm | 14911 | 129 | 1.05 (0.83-1.33) | 0.679 | 1.04 (0.82-1.31) | 0.755 | 1.05 (0.83-1.32) | 0.710 |
|  | RHR >80 bpm | 10584 | 113 | 1.33 (1.04-1.69) | 0.023 | 1.27 (0.99-1.62) | 0.056 | 1.26 (0.98-1.61) | 0.072 |
| Abbreviations: RHR, resting heart rate; ACD, all-cause dementia; AD, Alzheimer's disease; VaD, vascular dementia; HR, hazard ratios, 95CI%, 95% confidential intervals *Model 1: Adjusted for age, sex, education, and *APOE* 4 Status *Model 2: Adjusted for covariates of model 1 + smoking, physical activity, and BMI *Model 3: Adjusted for all covariates | | | | | | | | | |

| **SM 5 - Table 2b The associations between resting heart rate and dementia outcomes in age restricted models (Age<65)** | | | | | | | | | |
| --- | --- | --- | --- | --- | --- | --- | --- | --- | --- |
|  | **RHR** | **No. of participants** | **No. dementia cases** | **Model 1** | | **Model 2** | | **Model 3** | |
|  |  |  |  | **HR (95%CI)** | ***P*** | **HR (95%CI)** | ***P*** | **HR (95%CI)** | ***P*** |
|  | RHR per 10 bpm increment | 284482 | 2085 | 1.07 (1.04-1.11) | <0.001 | 1.07 (1.03-1.11) | <0.001 | 1.04 (1.00-1.11) | <0.001 |
|  | Categorical RHR |  |  |  |  |  |  |  |  |
| ACD | RHR <60 bpm | 53125 | 355 | 0.9 (0.8-1.03) | 0.123 | 0.91 (0.8-1.04) | 0.157 | 0.86 (0.76-0.98) | 0.025 |
|  | RHR 60-69 bpm | 100091 | 699 | Reference |  | Reference |  | Reference |  |
|  | RHR 70-79 bpm | 80227 | 582 | 1.01 (0.91-1.13) | 0.810 | 1 (0.89-1.11) | 0.978 | 1.01 (0.91-1.13) | 0.811 |
|  | RHR >80 bpm | 51039 | 449 | 1.16 (1.03-1.3) | 0.016 | 1.14 (1.01-1.29) | 0.031 | 1.11 (0.98-1.25) | 0.100 |
|  | RHR per 10 bpm increment | 283294 | 897 | 1.04 (0.98-1.1) | 0.167 | 1.05 (0.99-1.11) | 0.081 | 1.04 (0.98-1.1) | 0.153 |
|  | Categorical RHR |  |  |  |  |  |  |  |  |
| AD | RHR <60 bpm | 52913 | 143 | 0.85 (0.7-1.04) | 0.116 | 0.85 (0.7-1.04) | 0.108 | 0.84 (0.68-1.02) | 0.082 |
|  | RHR 60-69 bpm | 99696 | 304 | Reference |  | Reference |  | Reference |  |
|  | RHR 70-79 bpm | 79929 | 284 | 1.13 (0.96-1.33) | 0.142 | 1.14 (0.97-1.34) | 0.112 | 1.14 (0.97-1.34) | 0.115 |
|  | RHR >80 bpm | 50756 | 166 | 0.98 (0.81-1.19) | 0.854 | 1.02 (0.84-1.23) | 0.877 | 0.98 (0.81-1.19) | 0.871 |
|  | RHR per 10 bpm increment | 282873 | 476 | 1.11 (1.03-1.19) | 0.007 | 1.07 (1-1.15) | 0.064 | 1.09 (1.01-1.18) | 0.020 |
|  | Categorical RHR |  |  |  |  |  |  |  |  |
| VaD | RHR <60 bpm | 52870 | 100 | 1.22 (0.94-1.57) | 0.136 | 1.25 (0.96-1.61) | 0.095 | 1.09 (0.84-1.42) | 0.529 |
|  | RHR 60-69 bpm | 99532 | 140 | Reference |  | Reference |  | Reference |  |
|  | RHR 70-79 bpm | 79765 | 120 | 1.06 (0.83-1.35) | 0.652 | 1 (0.78-1.28) | 1.000 | 1.05 (0.82-1.34) | 0.720 |
|  | RHR >80 bpm | 50706 | 116 | 1.49 (1.16-1.9) | 0.002 | 1.37 (1.07-1.75) | 0.014 | 1.3 (1.01-1.68) | 0.041 |
| Abbreviations: RHR, resting heart rate; ACD, all-cause dementia; AD, Alzheimer's disease; VaD, vascular dementia; HR, hazard ratios, 95CI%, 95% confidential intervals *Model 1: Adjusted for age, sex, education, and *APOE* 4 Status *Model 2: Adjusted for covariates of model 1 + smoking, physical activity, and BMI *Model 3: Adjusted for all covariates | | | | | | | | | |

## Sex-restricted models

| **SM 5 – Table 3a Interaction terms for sex** | | | |
| --- | --- | --- | --- |
| **P for interaction** | **Model 1** | **Model 2** | **Model 3** |
| ACD | 0.0275 | 0.0404 | 0.0344 |
| AD | 0.8810 | 0.9382 | 0.9414 |
| VD | 0.0677 | 0.0759 | 0.0640 |
| Abbreviations: ACD, all-cause dementia; AD, Alzheimer's disease; VaD, vascular dementia *Model 1: Adjusted for age, sex, education, and *APOE* 4 Status *Model 2: Adjusted for covariates of model 1 + smoking, physical activity, and BMI *Model 3: Adjusted for all covariates | | | |

| **SM 5 - Table 3b The associations between resting heart rate and dementia outcomes in sex restricted models (Male)** | | | | | | | | | |
| --- | --- | --- | --- | --- | --- | --- | --- | --- | --- |
|  | **RHR** | **No. of participants** | **No. dementia cases** | **Model 1** | | **Model 2** | | **Model 3** | |
|  |  |  |  | **HR (95%CI)** | ***P*** | **HR (95%CI)** | ***P*** | **HR (95%CI)** | ***P*** |
|  | RHR per 10 bpm increment | 183006 | 1952 | 1.02 (0.98-1.06) | 0.256 | 1.02 (0.98-1.06) | 0.331 | 1.02 (0.98-1.06) | 0.264 |
|  | Categorical RHR |  |  |  |  |  |  |  |  |
| ACD | RHR <60 bpm | 26390 | 284 | 1.07 (0.93-1.24) | 0.313 | 1.08 (0.94-1.24) | 0.287 | 1.01 (0.88-1.17) | 0.871 |
|  | RHR 60-69 bpm | 65265 | 646 | Reference |  | Reference |  | Reference |  |
|  | RHR 70-79 bpm | 56573 | 577 | 0.97 (0.86-1.08) | 0.565 | 0.96 (0.86-1.08) | 0.530 | 0.97 (0.87-1.09) | 0.608 |
|  | RHR >80 bpm | 34778 | 445 | 1.1 (0.98-1.24) | 0.113 | 1.09 (0.97-1.24) | 0.146 | 1.06 (0.94-1.2) | 0.328 |
|  | RHR per 10 bpm increment | 182007 | 953 | 1.02 (0.96-1.08) | 0.541 | 1.02 (0.97-1.08) | 0.400 | 1.01 (0.96-1.07) | 0.665 |
|  | Categorical RHR |  |  |  |  |  |  |  |  |
| AD | RHR <60 bpm | 26237 | 131 | 1.06 (0.86-1.3) | 0.578 | 1.06 (0.87-1.31) | 0.555 | 1.03 (0.84-1.27) | 0.768 |
|  | RHR 60-69 bpm | 64921 | 302 | Reference |  | Reference |  | Reference |  |
|  | RHR 70-79 bpm | 56313 | 317 | 1.13 (0.96-1.32) | 0.130 | 1.18 (1.01-1.39) | 0.036 | 1.13 (0.97-1.33) | 0.123 |
|  | RHR >80 bpm | 34536 | 203 | 1.07 (0.9-1.28) | 0.454 | 1.09 (0.92-1.31) | 0.322 | 1.05 (0.87-1.25) | 0.629 |
|  | RHR per 10 bpm increment | 181448 | 394 | 1 (0.92-1.09) | 0.964 | 0.98 (0.9-1.07) | 0.643 | 1 (0.92-1.1) | 0.942 |
|  | Categorical RHR |  |  |  |  |  |  |  |  |
| VD | RHR <60 bpm | 26183 | 77 | 1.54 (1.16-2.04) | 0.003 | 1.55 (1.16-2.06) | 0.003 | 1.37 (1.02-1.84) | 0.037 |
|  | RHR 60-69 bpm | 64741 | 122 | Reference |  | Reference |  | Reference |  |
|  | RHR 70-79 bpm | 56097 | 101 | 0.89 (0.68-1.16) | 0.390 | 0.9 (0.69-1.17) | 0.411 | 0.89 (0.68-1.16) | 0.400 |
|  | RHR >80 bpm | 34427 | 94 | 1.23 (0.94-1.6) | 0.138 | 1.16 (0.89-1.52) | 0.281 | 1.13 (0.86-1.49) | 0.388 |
| Abbreviations: RHR, resting heart rate; ACD, all-cause dementia; AD, Alzheimer's disease; VaD, vascular dementia; HR, hazard ratios, 95CI%, 95% confidential intervals *Model 1: Adjusted for age, sex, education, and *APOE* 4 Status *Model 2: Adjusted for covariates of model 1 + smoking, physical activity, and BMI *Model 3: Adjusted for all covariates | | | | | | | | | |

| **SM 5 - Table 3c The associations between resting heart rate and dementia outcomes in sex restricted models (Female)** | | | | | | | | | |
| --- | --- | --- | --- | --- | --- | --- | --- | --- | --- |
|  | **RHR** | **No. of participants** | **No. dementia cases** | **Model 1** | | **Model 2** | | **Model 3** | |
|  |  |  |  | **HR (95%CI)** | ***P*** | **HR (95%CI)** | ***P*** | **HR (95%CI)** | ***P*** |
|  | RHR per 10 bpm increment | 156895 | 2225 | 1.08 (1.05-1.12) | <0.001 | 1.08 (1.04-1.11) | <0.001 | 1.09 (1.05-1.13) | <0.001 |
|  | Categorical RHR |  |  |  |  |  |  |  |  |
| ACD | RHR <60 bpm | 37321 | 500 | 0.98 (0.87-1.1) | 0.731 | 0.98 (0.88-1.11) | 0.796 | 0.93 (0.83-1.04) | 0.211 |
|  | RHR 60-69 bpm | 53385 | 695 | Reference |  | Reference |  | Reference |  |
|  | RHR 70-79 bpm | 38998 | 567 | 1.12 (1.01-1.26) | 0.038 | 1.12 (1-1.25) | 0.049 | 1.13 (1.01-1.26) | 0.034 |
|  | RHR >80 bpm | 27191 | 463 | 1.31 (1.16-1.47) | <0.001 | 1.3 (1.16-1.46) | <0.001 | 1.29 (1.14-1.45) | <0.001 |
|  | RHR per 10 bpm increment | 155554 | 884 | 1.02 (0.97-1.08) | 0.417 | 1.04 (0.98-1.09) | 0.205 | 1.04 (0.98-1.1) | 0.198 |
|  | Categorical RHR |  |  |  |  |  |  |  |  |
| AD | RHR <60 bpm | 37036 | 215 | 1.01 (0.84-1.2) | 0.951 | 1 (0.83-1.19) | 0.960 | 0.97 (0.81-1.17) | 0.779 |
|  | RHR 60-69 bpm | 52978 | 288 | Reference |  | Reference |  | Reference |  |
|  | RHR 70-79 bpm | 38655 | 224 | 1.08 (0.9-1.28) | 0.404 | 1.05 (0.88-1.25) | 0.586 | 1.09 (0.92-1.3) | 0.314 |
|  | RHR >80 bpm | 26885 | 157 | 1.08 (0.89-1.32) | 0.417 | 1.12 (0.92-1.37) | 0.243 | 1.11 (0.91-1.35) | 0.303 |
|  | RHR per 10 bpm increment | 155265 | 595 | 1.1 (1.04-1.17) | 0.002 | 1.08 (1.01-1.15) | 0.024 | 1.1 (1.03-1.17) | 0.003 |
|  | Categorical RHR |  |  |  |  |  |  |  |  |
| VaD | RHR <60 bpm | 36963 | 142 | 1.13 (0.9-1.41) | 0.280 | 1.16 (0.92-1.44) | 0.204 | 1.02 (0.81-1.28) | 0.882 |
|  | RHR 60-69 bpm | 52860 | 170 | Reference |  | Reference |  | Reference |  |
|  | RHR 70-79 bpm | 38579 | 148 | 1.2 (0.96-1.49) | 0.112 | 1.11 (0.89-1.39) | 0.341 | 1.18 (0.94-1.47) | 0.145 |
|  | RHR >80 bpm | 26863 | 135 | 1.55 (1.24-1.94) | <0.001 | 1.45 (1.15-1.82) | 0.001 | 1.41 (1.12-1.78) | 0.003 |
| Abbreviations: RHR, resting heart rate; ACD, all-cause dementia; AD, Alzheimer's disease; VaD, vascular dementia; HR, hazard ratios, 95CI%, 95% confidential intervals *Model 1: Adjusted for age, sex, education, and *APOE* 4 Status *Model 2: Adjusted for covariates of model 1 + smoking, physical activity, and BMI *Model 3: Adjusted for all covariates | | | | | | | | | |

## Follow-up-restricted models

| **SM 5 - Table 4 The associations between resting heart rate and dementia outcomes in follow-up restricted models** (**> 5 years)** | | | | | | | | | |
| --- | --- | --- | --- | --- | --- | --- | --- | --- | --- |
|  | **RHR** | **No. of participants** | **No. dementia Cases** | **Model 1** | | **Model 2** | | **Model 3** | |
|  |  |  |  | **HR (95%CI)** | ***P*** | **HR (95%CI)** | ***P*** | **HR (95%CI)** | ***P*** |
|  | RHR per 10 bpm increment | 311057 | 3588 | 1.05 (1.02-1.08) | <0.001 | 1.05 (1.02-1.08) | 0.001 | 1.06 (1.03-1.09) | <0.001 |
|  | Categorical RHR |  |  |  |  |  |  |  |  |
| ACD | RHR <60 bpm | 58379 | 662 | 0.97 (0.88-1.07) | 0.560 | 0.98 (0.89-1.08) | 0.643 | 0.92 (0.84-1.02) | 0.107 |
|  | RHR 60-69 bpm | 108859 | 1169 | Reference |  | Reference |  | Reference |  |
|  | RHR 70-79 bpm | 87533 | 995 | 1.04 (0.95-1.13) | 0.382 | 1.03 (0.95-1.12) | 0.445 | 1.04 (0.96-1.13) | 0.339 |
|  | RHR >80 bpm | 56286 | 762 | 1.16 (1.06-1.27) | 0.002 | 1.15 (1.05-1.26) | 0.003 | 1.13 (1.03-1.24) | 0.009 |
|  | RHR per 10 bpm increment | 309063 | 1594 | 1.04 (1-1.08) | 0.070 | 1.05 (1.01-1.09) | 0.027 | 1.04 (1-1.09) | 0.065 |
|  | Categorical RHR |  |  |  |  |  |  |  |  |
| AD | RHR <60 bpm | 58013 | 296 | 1.03 (0.89-1.19) | 0.680 | 1.03 (0.89-1.19) | 0.697 | 1.01 (0.87-1.17) | 0.861 |
|  | RHR 60-69 bpm | 108191 | 501 | Reference |  | Reference |  | Reference |  |
|  | RHR 70-79 bpm | 87013 | 475 | 1.14 (1.01-1.3) | 0.037 | 1.15 (1.02-1.31) | 0.025 | 1.15 (1.01-1.3) | 0.032 |
|  | RHR >80 bpm | 55846 | 322 | 1.13 (0.99-1.3) | 0.080 | 1.16 (1.01-1.34) | 0.034 | 1.13 (0.98-1.3) | 0.090 |
|  | RHR per 10 bpm increment | 308306 | 837 | 1.04 (0.99-1.1) | 0.153 | 1.02 (0.96-1.08) | 0.567 | 1.04 (0.98-1.1) | 0.198 |
|  | Categorical RHR |  |  |  |  |  |  |  |  |
| VaD | RHR <60 bpm | 57901 | 184 | 1.22 (1.01-1.48) | 0.042 | 1.24 (1.02-1.5) | 0.028 | 1.1 (0.91-1.34) | 0.326 |
|  | RHR 60-69 bpm | 107940 | 250 | Reference |  | Reference |  | Reference |  |
|  | RHR 70-79 bpm | 86760 | 222 | 1.09 (0.91-1.31) | 0.340 | 1.06 (0.89-1.27) | 0.513 | 1.08 (0.9-1.3) | 0.395 |
|  | RHR >80 bpm | 55705 | 181 | 1.29 (1.07-1.56) | 0.009 | 1.21 (1-1.47) | 0.054 | 1.18 (0.97-1.43) | 0.099 |
| Abbreviations: RHR, resting heart rate; ACD, all-cause dementia; AD, Alzheimer's disease; VaD, vascular dementia; HR, hazard ratios, 95CI%, 95% confidential intervals *Model 1: Adjusted for age, sex, education, and *APOE* 4 Status *Model 2: Adjusted for covariates of model 1 + smoking, physical activity, and BMI *Model 3: Adjusted for all covariates | | | | | | | | | |

## Heart rate reducing medication status-restricted models

| **SM 5 – Table 5a Interaction terms for heart rate reducing medication status** | | | |
| --- | --- | --- | --- |
| **P for interaction** | **Model 1** | **Model 2** | **Model 3** |
| ACD | 0.1820 | 0.1893 | 0.4705 |
| AD | 0.8730 | 0.9259 | 0.8939 |
| VD | 0.7688 | 0.7421 | 0.3798 |
| Abbreviations: ACD, all-cause dementia; AD, Alzheimer's disease; VaD, vascular dementia *Model 1: Adjusted for age, sex, education, and *APOE* 4 Status *Model 2: Adjusted for covariates of model 1 + smoking, physical activity, and BMI *Model 3: Adjusted for all covariates | | | |

| **SM 5 - Table 5b The associations between resting heart rate and dementia outcomes in group prescribing heart rate reducing medication** | | | | | | | | | |
| --- | --- | --- | --- | --- | --- | --- | --- | --- | --- |
|  | **RHR** | **No. of participants** | **No. dementia Cases** | **Model 1** | | **Model 2** | | **Model 3** | |
|  |  |  |  | **HR (95%CI)** | ***P*** | **HR (95%CI)** | ***P*** | **HR (95%CI)** | ***P*** |
|  | RHR per 10 bpm increment | 25921 | 695 | 1.12 (1.06-1.19) | <0.001 | 1.12 (1.05-1.19) | <0.001 | 1.09 (1.02-1.16) | 0.008 |
|  | Categorical RHR |  |  |  |  |  |  |  |  |
| ACD | RHR <60 bpm | 11291 | 282 | 0.89 (0.75-1.07) | 0.208 | 0.9 (0.75-1.07) | 0.234 | 0.93 (0.78-1.11) | 0.411 |
|  | RHR 60-69 bpm | 8484 | 219 | Reference |  | Reference |  | Reference |  |
|  | RHR 70-79 bpm | 3907 | 125 | 1.32 (1.06-1.65) | 0.012 | 1.31 (1.05-1.64) | 0.015 | 1.28 (1.03-1.59) | 0.029 |
|  | RHR >80 bpm | 2239 | 69 | 1.28 (0.98-1.68) | 0.076 | 1.27 (0.97-1.66) | 0.085 | 1.19 (0.91-1.57) | 0.210 |
|  | RHR per 10 bpm increment | 25482 | 256 | 1.04 (0.93-1.16) | 0.479 | 1.03 (0.93-1.15) | 0.529 | 1 (0.9-1.12) | 0.971 |
|  | Categorical RHR |  |  |  |  |  |  |  |  |
| AD | RHR <60 bpm | 11117 | 108 | 0.87 (0.66-1.16) | 0.354 | 0.88 (0.66-1.17) | 0.365 | 0.91 (0.68-1.21) | 0.505 |
|  | RHR 60-69 bpm | 8350 | 85 | Reference |  | Reference |  | Reference |  |
|  | RHR 70-79 bpm | 3831 | 49 | 1.34 (0.95-1.91) | 0.099 | 1.33 (0.93-1.89) | 0.113 | 1.28 (0.9-1.82) | 0.172 |
|  | RHR >80 bpm | 2184 | 14 | 0.68 (0.39-1.2) | 0.184 | 0.68 (0.39-1.19) | 0.179 | 0.62 (0.35-1.1) | 0.103 |
|  | RHR per 10 bpm increment | 25458 | 232 | 2.74 (0.95-7.86) | 0.061 | 2.44 (0.84-7.13) | 0.102 | 1.71 (0.57-5.09) | 0.337 |
|  | Categorical RHR |  |  |  |  |  |  |  |  |
| VaD | RHR <60 bpm | 11111 | 102 | 1.08 (0.79-1.48) | 0.626 | 1.1 (0.8-1.5) | 0.559 | 1.15 (0.84-1.57) | 0.390 |
|  | RHR 60-69 bpm | 8329 | 64 | Reference |  | Reference |  | Reference |  |
|  | RHR 70-79 bpm | 3824 | 42 | 1.51 (1.02-2.22) | 0.039 | 1.48 (1-2.18) | 0.050 | 1.42 (0.96-2.1) | 0.078 |
|  | RHR >80 bpm | 2194 | 24 | 1.49 (0.93-2.39) | 0.094 | 1.46 (0.92-2.34) | 0.112 | 1.34 (0.84-2.16) | 0.222 |
| Abbreviations: RHR, resting heart rate; ACD, all-cause dementia; AD, Alzheimer's disease; VaD, vascular dementia; HR, hazard ratios, 95CI%, 95% confidential intervals *Model 1: Adjusted for age, sex, education, and *APOE* 4 Status *Model 2: Adjusted for covariates of model 1 + smoking, physical activity, and BMI *Model 3: Adjusted for all covariates | | | | | | | | | |

| **SM 5 - Table 5c The associations between resting heart rate and dementia outcomes in group not prescribing heart rate reducing medication** | | | | | | | | | |
| --- | --- | --- | --- | --- | --- | --- | --- | --- | --- |
|  | **RHR** | **No. of participants** | **No. dementia Cases** | **Model 1** | | **Model 2** | | **Model 3** | |
|  |  |  |  | **HR (95%CI)** | ***P*** | **HR (95%CI)** | ***P*** | **HR (95%CI)** | ***P*** |
|  | RHR per 10 bpm increment | 313980 | 3482 | 1.07 (1.05-1.1) | <0.001 | 1.07 (1.04-1.1) | <0.001 | 1.06 (1.03-1.09) | <0.001 |
|  | Categorical RHR |  |  |  |  |  |  |  |  |
| ACD | RHR <60 bpm | 52420 | 502 | 0.97 (0.88-1.08) | 0.609 | 0.98 (0.88-1.09) | 0.676 | 0.97 (0.88-1.08) | 0.617 |
|  | RHR 60-69 bpm | 110166 | 1122 | Reference |  | Reference |  | Reference |  |
|  | RHR 70-79 bpm | 91664 | 1019 | 1.04 (0.96-1.13) | 0.340 | 1.04 (0.96-1.13) | 0.349 | 1.03 (0.94-1.12) | 0.569 |
|  | RHR >80 bpm | 59730 | 839 | 1.23 (1.13-1.35) | <0.001 | 1.24 (1.13-1.35) | <0.001 | 1.18 (1.07-1.29) | 0.001 |
|  | RHR per 10 bpm increment | 312079 | 1581 | 1.03 (0.99-1.07) | 0.185 | 1.04 (1-1.09) | 0.044 | 1.03 (0.99-1.07) | 0.181 |
|  | Categorical RHR |  |  |  |  |  |  |  |  |
| AD | RHR <60 bpm | 52156 | 238 | 1.05 (0.9-1.22) | 0.544 | 1.04 (0.89-1.21) | 0.660 | 1.04 (0.89-1.21) | 0.634 |
|  | RHR 60-69 bpm | 109549 | 505 | Reference |  | Reference |  | Reference |  |
|  | RHR 70-79 bpm | 91137 | 492 | 1.1 (0.97-1.25) | 0.121 | 1.12 (0.99-1.27) | 0.069 | 1.11 (0.98-1.26) | 0.106 |
|  | RHR >80 bpm | 59237 | 346 | 1.12 (0.98-1.29) | 0.097 | 1.17 (1.02-1.34) | 0.026 | 1.12 (0.98-1.29) | 0.101 |
|  | RHR per 10 bpm increment | 311255 | 757 | 3.61 (2.03-6.42) | <0.001 | 2.85 (1.58-5.14) | <0.001 | 1.94 (1.07-3.51) | 0.028 |
|  | Categorical RHR |  |  |  |  |  |  |  |  |
| VaD | RHR <60 bpm | 52035 | 117 | 1.08 (0.86-1.35) | 0.510 | 1.11 (0.89-1.39) | 0.364 | 1.1 (0.88-1.37) | 0.419 |
|  | RHR 60-69 bpm | 109272 | 228 | Reference |  | Reference |  | Reference |  |
|  | RHR 70-79 bpm | 90852 | 207 | 1.05 (0.87-1.27) | 0.603 | 1.02 (0.85-1.24) | 0.817 | 0.98 (0.82-1.19) | 0.873 |
|  | RHR >80 bpm | 59096 | 205 | 1.49 (1.24-1.8) | <0.001 | 1.4 (1.16-1.7) | 0.001 | 1.25 (1.03-1.52) | 0.023 |
| Abbreviations: RHR, resting heart rate; ACD, all-cause dementia; AD, Alzheimer's disease; VaD, vascular dementia; HR, hazard ratios, 95CI%, 95% confidential intervals *Model 1: Adjusted for age, sex, education, and *APOE* 4 Status *Model 2: Adjusted for covariates of model 1 + smoking, physical activity, and BMI *Model 3: Adjusted for all covariates | | | | | | | | | |

# Supplementary Materials 6 - Sensitivity analyses of cognitive decline

## CVD-restricted models

| **SM 6 – Table 1 The associations between resting heart rate and cognitive decline in CVD-restricted models** | | | | | | | |
| --- | --- | --- | --- | --- | --- | --- | --- |
|  | **RHR** | **Model 1** | | **Model 2** | | **Model 3** | |
|  |  | **OR (95%CI)** | **FDR - *P*** | **OR (95%CI)** | **FDR - *P*** | **OR (95%CI)** | **FDR - *P*** |
| **Fluid intelligence** | RHR per 10 bpm increment | 1.063 (1.02-1.1) | 0.001 | 1.015 (1.01-1.02) | 0.001 | 1.016 (1.01-1.03) | <0.001 |
|  | Categorical RHR |  |  |  |  |  |  |
|  | RHR <60 bpm | 0.887 (0.8-0.98) | 0.046 | 0.972 (0.95-1) | 0.044 | 0.97 (0.95-0.99) | 0.032 |
|  | RHR 60-69 bpm | Reference |  | Reference |  | Reference |  |
|  | RHR 70-79 bpm | 0.967 (0.88-1.07) | 0.508 | 0.992 (0.97-1.02) | 0.520 | 0.994 (0.97-1.02) | 0.598 |
|  | RHR >80 bpm | 1.112 (0.98-1.26) | 0.093 | 1.026 (1-1.06) | 0.091 | 1.028 (1-1.06) | 0.070 |
| **Reaction time** | RHR per 10 bpm increment | 1.063 (1.04-1.09) | <0.001 | 1.013 (1.01-1.02) | <0.001 | 1.013 (1.01-1.02) | <0.001 |
|  | Categorical RHR |  |  |  |  |  |  |
|  | RHR <60 bpm | 0.954 (0.89-1.02) | 0.182 | 0.99 (0.98-1) | 0.164 | 0.99 (0.98-1) | 0.165 |
|  | RHR 60-69 bpm | Reference |  | Reference |  | Reference |  |
|  | RHR 70-79 bpm | 1.128 (1.06-1.2) | <0.001 | 1.025 (1.01-1.04) | <0.001 | 1.025 (1.01-1.04) | <0.001 |
|  | RHR >80 bpm | 1.147 (1.06-1.24) | 0.002 | 1.029 (1.01-1.05) | 0.002 | 1.029 (1.01-1.05) | 0.002 |
| Abbreviations: RHR, resting heart rate; HR, hazard ratios, 95CI%, 95% confidential intervals *Model 1: Adjusted for age, sex, education, and *APOE* 4 Status *Model 2: Adjusted for covariates of model 1 + smoking, physical activity, and BMI *Model 3: Adjusted for all covariates | | | | | | | |

## Age-restricted models

| **SM 6 - Table 2a The associations between resting heart rate and cognitive decline in age-restricted models** (**Age<65)** | | | | | | | |
| --- | --- | --- | --- | --- | --- | --- | --- |
|  | **RHR** | **Model 1** | | **Model 2** | | **Model 3** | |
|  |  | **OR (95%CI)** | **FDR - *P*** | **OR (95%CI)** | **FDR - *P*** | **OR (95%CI)** | **FDR - *P*** |
| **Fluid intelligence** | RHR per 10 bpm increment | 1.06 (1.02-1.1) | 0.002 | 1.014 (1.01-1.02) | 0.002 | 1.015 (1.01-1.02) | 0.001 |
|  | Categorical RHR |  |  |  |  |  |  |
|  | RHR <60 bpm | 0.908 (0.82-1.01) | 0.146 | 0.977 (0.95-1) | 0.148 | 0.976 (0.95-1) | 0.112 |
|  | RHR 60-69 bpm | Reference |  | Reference |  | Reference |  |
|  | RHR 70-79 bpm | 0.978 (0.88-1.08) | 0.677 | 0.995 (0.97-1.02) | 0.678 | 0.996 (0.97-1.02) | 0.728 |
|  | RHR >80 bpm | 1.112 (0.98-1.26) | 0.108 | 1.026 (0.99-1.06) | 0.111 | 1.027 (1-1.06) | 0.099 |
| **Reaction time** | RHR per 10 bpm increment | 1.062 (1.04-1.09) | <0.001 | 1.012 (1.01-1.02) | <0.001 | 1.013 (1.01-1.02) | <0.001 |
|  | Categorical RHR |  |  |  |  |  |  |
|  | RHR <60 bpm | 0.954 (0.89-1.02) | 0.196 | 0.991 (0.98-1) | 0.194 | 0.989 (0.98-1) | 0.133 |
|  | RHR 60-69 bpm | Reference |  | Reference |  | Reference |  |
|  | RHR 70-79 bpm | 1.109 (1.04-1.18) | 0.004 | 1.021 (1.01-1.03) | 0.004 | 1.022 (1.01-1.04) | 0.004 |
|  | RHR >80 bpm | 1.161 (1.07-1.26) | <0.001 | 1.03 (1.01-1.05) | <0.001 | 1.031 (1.01-1.05) | <0.001 |
| Abbreviations: RHR, resting heart rate; HR, hazard ratios, 95CI%, 95% confidential intervals *Model 1: Adjusted for age, sex, education, and *APOE* 4 Status *Model 2: Adjusted for covariates of model 1 + smoking, physical activity, and BMI *Model 3: Adjusted for all covariates | | | | | | | |

| **SM 6 - Table 2b The associations between resting heart rate and cognitive decline in age-restricted models** (**Age>65)** | | | | | | | |
| --- | --- | --- | --- | --- | --- | --- | --- |
|  | **RHR** | **Model 1** | | **Model 2** | | **Model 3** | |
|  |  | **OR (95%CI)** | **FDR - *P*** | **OR (95%CI)** | **FDR - *P*** | **OR (95%CI)** | **FDR - *P*** |
| **Fluid intelligence** | RHR per 10 bpm increment | 1.062 (0.94-1.2) | 0.328 | 1.014 (0.98-1.04) | 0.369 | 1.015 (0.98-1.05) | 0.350 |
|  | Categorical RHR |  |  |  |  |  |  |
|  | RHR <60 bpm | 0.731 (0.51-1.05) | 0.178 | 0.931 (0.85-1.02) | 0.216 | 0.93 (0.85-1.02) | 0.220 |
|  | RHR 60-69 bpm | Reference |  | Reference |  | Reference |  |
|  | RHR 70-79 bpm | 0.817 (0.59-1.14) | 0.230 | 0.951 (0.88-1.03) | 0.226 | 0.956 (0.88-1.04) | 0.281 |
|  | RHR >80 bpm | 1.161 (0.78-1.74) | 0.932 | 1.038 (0.94-1.15) | 0.940 | 1.038 (0.94-1.15) | 0.946 |
| **Reaction time** | RHR per 10 bpm increment | 1.071 (0.99-1.16) | 0.162 | 1.018 (1-1.04) | 0.090 | 1.015 (1-1.03) | 0.188 |
|  | Categorical RHR |  |  |  |  |  |  |
|  | RHR <60 bpm | 0.89 (0.7-1.13) | 0.347 | 0.973 (0.92-1.03) | 0.320 | 0.979 (0.93-1.03) | 0.431 |
|  | RHR 60-69 bpm | Reference |  | Reference |  | Reference |  |
|  | RHR 70-79 bpm | 1.241 (1.01-1.53) | 0.084 | 1.054 (1-1.1) | 0.060 | 1.051 (1-1.1) | 0.076 |
|  | RHR >80 bpm | 1.023 (0.79-1.32) | 0.864 | 1.011 (0.95-1.07) | 0.717 | 1.007 (0.95-1.07) | 0.809 |
| Abbreviations: RHR, resting heart rate; HR, hazard ratios, 95CI%, 95% confidential intervals *Model 1: Adjusted for age, sex, education, and *APOE* 4 Status *Model 2: Adjusted for covariates of model 1 + smoking, physical activity, and BMI *Model 3: Adjusted for all covariates | | | | | | | |

## Sex-restricted models

| **SM 6 - Table 3a The associations between resting heart rate and cognitive decline in sex-restricted models (Male)** | | | | | | | |
| --- | --- | --- | --- | --- | --- | --- | --- |
|  | **RHR** | **Model 1** | | **Model 2** | | **Model 3** | |
|  |  | **OR (95%CI)** | **FDR - *P*** | **OR (95%CI)** | **FDR - *P*** | **OR (95%CI)** | **FDR - *P*** |
| **Fluid intelligence** | RHR per 10 bpm increment | 1.075 (1.02-1.13) | 0.006 | 1.019 (1.01-1.03) | 0.005 | 1.021 (1.01-1.03) | 0.002 |
|  | Categorical RHR |  |  |  |  |  |  |
|  | RHR <60 bpm | 0.88 (0.76-1.02) | 0.196 | 0.969 (0.93-1) | 0.168 | 0.965 (0.93-1) | 0.112 |
|  | RHR 60-69 bpm | Reference |  | Reference |  | Reference |  |
|  | RHR 70-79 bpm | 0.944 (0.83-1.08) | 0.399 | 0.987 (0.96-1.02) | 0.425 | 0.989 (0.96-1.02) | 0.507 |
|  | RHR >80 bpm | 1.16 (0.98-1.37) | 0.080 | 1.038 (1-1.08) | 0.070 | 1.042 (1-1.08) | 0.050 |
| **Reaction time** | RHR per 10 bpm increment | 1.059 (1.02-1.09) | 0.002 | 1.012 (1-1.02) | 0.002 | 1.013 (1.01-1.02) | 0.002 |
|  | Categorical RHR |  |  |  |  |  |  |
|  | RHR <60 bpm | 0.995 (0.9-1.1) | 0.924 | 0.999 (0.98-1.02) | 0.916 | 0.998 (0.98-1.02) | 0.818 |
|  | RHR 60-69 bpm | Reference |  | Reference |  | Reference |  |
|  | RHR 70-79 bpm | 1.138 (1.05-1.24) | 0.004 | 1.027 (1.01-1.04) | 0.006 | 1.028 (1.01-1.05) | 0.004 |
|  | RHR >80 bpm | 1.205 (1.09-1.33) | <0.001 | 1.039 (1.02-1.06) | 0.002 | 1.041 (1.02-1.06) | <0.001 |
| Abbreviations: RHR, resting heart rate; HR, hazard ratios, 95CI%, 95% confidential intervals *Model 1: Adjusted for age, sex, education, and *APOE* 4 Status *Model 2: Adjusted for covariates of model 1 + smoking, physical activity, and BMI *Model 3: Adjusted for all covariates | | | | | | | |

| **SM 6 – Table 3b The associations between resting heart rate and cognitive decline in sex-restricted models** (**Female)** | | | | | | | |
| --- | --- | --- | --- | --- | --- | --- | --- |
|  | **RHR** | **Model 1** | | **Model 2** | | **Model 3** | |
|  |  | **OR (95%CI)** | **FDR - *P*** | **OR (95%CI)** | **FDR - *P*** | **OR (95%CI)** | **FDR - *P*** |
| **Fluid intelligence** | RHR per 10 bpm increment | 1.045 (1-1.1) | 0.069 | 1.009 (1-1.02) | 0.121 | 1.009 (1-1.02) | 0.137 |
|  | Categorical RHR |  |  |  |  |  |  |
|  | RHR <60 bpm | 0.901 (0.79-1.03) | 0.134 | 0.977 (0.95-1.01) | 0.162 | 0.978 (0.95-1.01) | 0.171 |
|  | RHR 60-69 bpm | Reference |  | Reference |  | Reference |  |
|  | RHR 70-79 bpm | 0.98 (0.85-1.13) | 0.784 | 0.993 (0.96-1.03) | 0.707 | 0.993 (0.96-1.03) | 0.712 |
|  | RHR >80 bpm | 1.061 (0.89-1.27) | 0.520 | 1.011 (0.97-1.06) | 0.611 | 1.011 (0.97-1.06) | 0.635 |
| **Reaction time** | RHR per 10 bpm increment | 1.066 (1.03-1.1) | <0.001 | 1.013 (1.01-1.02) | <0.001 | 1.014 (1.01-1.02) | <0.001 |
|  | Categorical RHR |  |  |  |  |  |  |
|  | RHR <60 bpm | 0.91 (0.83-1) | 0.084 | 0.981 (0.96-1) | 0.076 | 0.98 (0.96-1) | 0.058 |
|  | RHR 60-69 bpm | Reference |  | Reference |  | Reference |  |
|  | RHR 70-79 bpm | 1.101 (1-1.21) | 0.080 | 1.02 (1-1.04) | 0.076 | 1.02 (1-1.04) | 0.076 |
|  | RHR >80 bpm | 1.078 (0.96-1.21) | 0.392 | 1.016 (0.99-1.04) | 0.382 | 1.014 (0.99-1.04) | 0.482 |
| Abbreviations: RHR, resting heart rate; HR, hazard ratios, 95CI%, 95% confidential intervals *Model 1: Adjusted for age, sex, education, and *APOE* 4 Status *Model 2: Adjusted for covariates of model 1 + smoking, physical activity, and BMI *Model 3: Adjusted for all covariates | | | | | | | |

## Follow-up-restricted models

| **SM 6 – Table 4 The associations between resting heart rate and cognitive decline in follow-up restricted models** (**> 5 years)** | | | | | | | |
| --- | --- | --- | --- | --- | --- | --- | --- |
|  | **RHR** | **Model 1** | | **Model 2** | | **Model 3** | |
|  |  | **OR (95%CI)** | **FDR - *P*** | **OR (95%CI)** | **FDR - *P*** | **OR (95%CI)** | **FDR - *P*** |
| **Fluid intelligence** | RHR per 10 bpm increment | 1.058 (1.02-1.1) | 0.002 | 1.014 (1-1.02) | 0.002 | 1.015 (1.01-1.02) | 0.002 |
|  | Categorical RHR |  |  |  |  |  |  |
|  | RHR <60 bpm | 0.915 (0.82-1.02) | 0.196 | 0.979 (0.95-1) | 0.188 | 0.977 (0.95-1) | 0.076 |
|  | RHR 60-69 bpm | Reference |  | Reference |  | Reference |  |
|  | RHR 70-79 bpm | 0.987 (0.89-1.09) | 0.799 | 0.997 (0.97-1.02) | 0.070 | 0.998 (0.97-1.02) | 0.854 |
|  | RHR >80 bpm | 1.125 (0.99-1.28) | 0.070 | 1.029 (1-1.06) | <0.001 | 1.03 (1-1.06) | 0.065 |
| **Reaction time** | RHR per 10 bpm increment | 1.066 (1.04-1.09) | <0.001 | 1.013 (1.01-1.02) | <0.001 | 1.014 (1.01-1.02) | <0.001 |
|  | Categorical RHR |  |  |  |  |  |  |
|  | RHR <60 bpm | 0.945 (0.88-1.01) | 0.117 | 0.988 (0.97-1) | 0.109 | 0.987 (0.97-1) | 0.136 |
|  | RHR 60-69 bpm | Reference |  | Reference |  | Reference |  |
|  | RHR 70-79 bpm | 1.123 (1.05-1.2) | <0.001 | 1.024 (1.01-1.04) | <0.001 | 1.025 (1.01-1.04) | <0.001 |
|  | RHR >80 bpm | 1.149 (1.06-1.24) | 0.002 | 1.029 (1.01-1.05) | 0.001 | 1.029 (1.01-1.05) | 0.002 |
| Abbreviations: RHR, resting heart rate; HR, hazard ratios, 95CI%, 95% confidential intervals *Model 1: Adjusted for age, sex, education, and *APOE* 4 Status *Model 2: Adjusted for covariates of model 1 + smoking, physical activity, and BMI *Model 3: Adjusted for all covariates | | | | | | | |

# Supplementary Materials 7 – Brain imaging analyses

## The associations between continuous measures of resting heart rate and hippocampal subfield volumes

| **SM 7 Table 1 - The associations between continuous measures of resting heart rate and hippocampal subfield volumes** | | | | |
| --- | --- | --- | --- | --- |
| **Hippocampal volume** | **β - coefficients** | **Standard Error** | ***P*** | **FDR - *P*** |
| **Left hemisphere** |  |  |  |  |
| CA1-body | -0.0064097 | 0.0061259 | 0.295 | 0.537 |
| CA1-head | -0.0089513 | 0.0057891 | 0.122 | 0.349 |
| CA3-body | -0.0080002 | 0.0062690 | 0.202 | 0.465 |
| CA3-head | -0.0252585 | 0.0059517 | <0.001 | <0.001 |
| CA4-body | -0.0024634 | 0.0060473 | 0.684 | 0.787 |
| CA4-head | -0.0170791 | 0.0057756 | 0.003 | 0.018 |
| Fimbria | -0.0301136 | 0.0060391 | <0.001 | <0.001 |
| GC-ML-DG-body | -0.0102989 | 0.0058971 | 0.081 | 0.252 |
| GC-ML-DG-head | -0.0213488 | 0.0057723 | <0.001 | 0.002 |
| Hata | -0.0243349 | 0.0059820 | <0.001 | <0.001 |
| Hippocampal-fissure | 0.0144860 | 0.0059871 | 0.016 | 0.062 |
| Hippocampal-tail | 0.0188036 | 0.0059304 | 0.002 | 0.010 |
| Paralaminar-nucleus | 0.0049752 | 0.0056968 | 0.382 | 0.572 |
| Presubiculum-body | 0.0055260 | 0.0059249 | 0.351 | 0.562 |
| Presubiculum-head | -0.0016607 | 0.0058511 | 0.777 | 0.863 |
| Subiculum-body | <0.0017747 | 0.0059498 | 0.896 | 0.929 |
| Subiculum-head | -0.0074142 | 0.0059341 | 0.212 | 0.465 |
| Whole-hippocampal-body | -0.0055535 | 0.0056871 | 0.329 | 0.548 |
| Whole-hippocampal-head | -0.0132261 | 0.0056143 | 0.018 | 0.067 |
| Whole-hippocampus | -0.0063574 | 0.0055574 | 0.253 | 0.489 |
| **Right hemisphere** |  |  |  |  |
| CA1-body | -0.0061238 | 0.0061393 | 0.319 | 0.548 |
| CA1-head | -0.0047702 | 0.0058447 | 0.414 | 0.572 |
| CA3-body | -0.0014418 | 0.0062005 | 0.816 | 0.882 |
| CA3-head | -0.0103935 | 0.0059728 | 0.082 | 0.252 |
| CA4-body | <0.0017120 | 0.0060365 | 0.906 | 0.929 |
| CA4-head | -0.0027360 | 0.0058583 | 0.640 | 0.787 |
| Fimbria | -0.0264912 | 0.0060182 | <0.001 | <0.001 |
| GC-ML-DG-body | -0.0050618 | 0.0059220 | 0.393 | 0.572 |
| GC-ML-DG-head | -0.0077635 | 0.0058696 | 0.186 | 0.465 |
| Hata | -0.0171465 | 0.0060400 | 0.005 | 0.020 |
| Hippocampal-fissure | 0.0167622 | 0.0058877 | 0.004 | 0.020 |
| Hippocampal-tail | 0.0087072 | 0.0059104 | 0.141 | 0.375 |
| Paralaminar-nucleus | -0.0028228 | 0.0057460 | 0.623 | 0.787 |
| Presubiculum-body | 0.0071460 | 0.0058350 | 0.221 | 0.465 |
| Presubiculum-head | -0.0039903 | 0.0058842 | 0.498 | 0.664 |
| Subiculum-body | -<0.0015295 | 0.0059511 | 0.929 | 0.929 |
| Subiculum-head | -0.0023943 | 0.0059810 | 0.689 | 0.787 |
| Whole-hippocampal-body | -0.0047780 | 0.0057047 | 0.402 | 0.572 |
| Whole-hippocampal-head | -0.0064307 | 0.0056689 | 0.257 | 0.489 |
| Whole-hippocampus | -0.0022900 | 0.0055935 | 0.682 | 0.787 |

## The associations between categorical measures of resting heart rate and hippocampal subfield volumes

| **SM 7 Table 1 - The associations between categorical measures of resting heart rate and hippocampal subfield volumes** | | | | | |
| --- | --- | --- | --- | --- | --- |
| **Hippocampal volume** | **RHR** | **β - coefficients** | **Standard Error** | ***P*** | **FDR - *P*** |
| **Left hemisphere** |  |  |  |  |  |
| CA1-body | RHR <60 bpm | -0.0106429 | 0.0159278 | 0.504 | 0.695 |
|  | RHR 70-79 bpm | -0.0153966 | 0.0148495 | 0.300 | 0.610 |
|  | RHR >80bpm | -0.0175045 | 0.0187478 | 0.350 | 0.610 |
| CA1-head | RHR <60 bpm | -0.0342798 | 0.0150440 | 0.023 | 0.210 |
|  | RHR 70-79 bpm | -0.0451963 | 0.0177189 | 0.011 | 0.108 |
|  | RHR >80bpm | -0.0239110 | 0.0140294 | 0.088 | 0.379 |
| CA3-body | RHR <60 bpm | -0.0187101 | 0.0162964 | 0.251 | 0.610 |
|  | RHR 70-79 bpm | -0.0260029 | 0.0151986 | 0.087 | 0.379 |
|  | RHR >80bpm | -0.0195217 | 0.0191826 | 0.309 | 0.610 |
| CA3-head | RHR <60 bpm | -0.0138486 | 0.0154665 | 0.371 | 0.610 |
|  | RHR 70-79 bpm | -0.0266438 | 0.0144255 | 0.065 | 0.370 |
|  | RHR >80bpm | -0.0792788 | 0.0182231 | <0.001 | 0.001 |
| CA4-body | RHR <60 bpm | -0.0170360 | 0.0157242 | 0.279 | 0.610 |
|  | RHR 70-79 bpm | -0.0108421 | 0.0146603 | 0.460 | 0.664 |
|  | RHR >80bpm | -0.0139920 | 0.0184945 | 0.449 | 0.658 |
| CA4-head | RHR <60 bpm | -0.0214486 | 0.0150086 | 0.153 | 0.540 |
|  | RHR 70-79 bpm | -0.0264430 | 0.0140016 | 0.059 | 0.354 |
|  | RHR >80bpm | -0.0622100 | 0.0176784 | <0.001 | 0.009 |
| Fimbria | RHR <60 bpm | 0.0049453 | 0.0156988 | 0.753 | 0.854 |
|  | RHR 70-79 bpm | -0.0285121 | 0.0146382 | 0.051 | 0.325 |
|  | RHR >80bpm | -0.0917181 | 0.0184862 | <0.001 | <0.001 |
| GC-ML-DG-body | RHR <60 bpm | -0.0054635 | 0.0153276 | 0.722 | 0.833 |
|  | RHR 70-79 bpm | -0.0150960 | 0.0142960 | 0.291 | 0.610 |
|  | RHR >80bpm | -0.0325864 | 0.0180402 | 0.071 | 0.379 |
| GC-ML-DG-head | RHR <60 bpm | -0.0133066 | 0.0150069 | 0.375 | 0.610 |
|  | RHR 70-79 bpm | -0.0245526 | 0.0139928 | 0.079 | 0.379 |
|  | RHR >80bpm | -0.0687950 | 0.0176702 | <0.001 | 0.004 |
| Hata | RHR <60 bpm | 0.0087820 | 0.0155482 | 0.572 | 0.738 |
|  | RHR 70-79 bpm | -0.0158824 | 0.0145021 | 0.273 | 0.610 |
|  | RHR >80bpm | -0.0650361 | 0.0183133 | <0.001 | 0.009 |
| Hippocampal-fissure | RHR <60 bpm | -0.0194733 | 0.0155569 | 0.211 | 0.599 |
|  | RHR 70-79 bpm | 0.0085020 | 0.0145127 | 0.558 | 0.736 |
|  | RHR >80bpm | 0.0320827 | 0.0183217 | 0.080 | 0.379 |
| Hippocampal-tail | RHR <60 bpm | -0.0468053 | 0.0154129 | 0.002 | 0.041 |
|  | RHR 70-79 bpm | -<0.0019057 | 0.0143750 | 0.950 | 0.966 |
|  | RHR >80bpm | 0.0230800 | 0.0181402 | 0.203 | 0.597 |
| Paralaminar-nucleus | RHR <60 bpm | -0.0438385 | 0.0147986 | 0.003 | 0.046 |
|  | RHR 70-79 bpm | -0.0049936 | 0.0138102 | 0.718 | 0.833 |
|  | RHR >80bpm | -0.0158065 | 0.0174353 | 0.365 | 0.610 |
| Presubiculum-body | RHR <60 bpm | -0.0154727 | 0.0154054 | 0.315 | 0.610 |
|  | RHR 70-79 bpm | -0.0060101 | 0.0143629 | 0.676 | 0.818 |
|  | RHR >80bpm | 0.0182973 | 0.0181136 | 0.312 | 0.610 |
| Presubiculum-head | RHR <60 bpm | -0.0324888 | 0.0152040 | 0.033 | 0.236 |
|  | RHR 70-79 bpm | -0.0096546 | 0.0141822 | 0.496 | 0.695 |
|  | RHR >80bpm | -0.0154707 | 0.0179071 | 0.388 | 0.618 |
| Subiculum-body | RHR <60 bpm | -0.0105390 | 0.0154668 | 0.496 | 0.695 |
|  | RHR 70-79 bpm | -0.0022164 | 0.0144179 | 0.878 | 0.925 |
|  | RHR >80bpm | 0.0019826 | 0.0182122 | 0.913 | 0.937 |
| Subiculum-head | RHR <60 bpm | -0.0188731 | 0.0154193 | 0.221 | 0.600 |
|  | RHR 70-79 bpm | -0.0141209 | 0.0143821 | 0.326 | 0.610 |
|  | RHR >80bpm | -0.0242445 | 0.0181657 | 0.182 | 0.590 |
| Whole-hippocampal-body | RHR <60 bpm | -0.0134149 | 0.0147826 | 0.364 | 0.610 |
|  | RHR 70-79 bpm | -0.0137599 | 0.0137820 | 0.318 | 0.610 |
|  | RHR >80bpm | -0.0154103 | 0.0174087 | 0.376 | 0.610 |
| Whole-hippocampal-head | RHR <60 bpm | -0.0230587 | 0.0145885 | 0.114 | 0.441 |
|  | RHR 70-79 bpm | -0.0176151 | 0.0136096 | 0.196 | 0.597 |
|  | RHR >80bpm | -0.0488529 | 0.0171857 | 0.004 | 0.060 |
| Whole-hippocampus | RHR <60 bpm | -0.0259061 | 0.0144420 | 0.073 | 0.379 |
|  | RHR 70-79 bpm | -0.0153733 | 0.0134707 | 0.254 | 0.610 |
|  | RHR >80bpm | -0.0282273 | 0.0170035 | 0.097 | 0.401 |
| **Right hemisphere** |  |  |  |  |  |
| CA1-body | RHR <60 bpm | -0.0162885 | 0.0159650 | 0.308 | 0.610 |
|  | RHR 70-79 bpm | -0.0040914 | 0.0148785 | 0.783 | 0.870 |
|  | RHR >80bpm | -0.0414772 | 0.0188021 | 0.027 | 0.219 |
| CA1-head | RHR <60 bpm | -0.0175000 | 0.0151918 | 0.249 | 0.610 |
|  | RHR 70-79 bpm | -0.0035930 | 0.0141719 | 0.800 | 0.874 |
|  | RHR >80bpm | -0.0144891 | 0.0178762 | 0.418 | 0.643 |
| CA3-body | RHR <60 bpm | -0.0091712 | 0.0161242 | 0.570 | 0.738 |
|  | RHR 70-79 bpm | 0.0072563 | 0.0150288 | 0.629 | 0.780 |
|  | RHR >80bpm | -0.0122991 | 0.0189794 | 0.517 | 0.705 |
| CA3-head | RHR <60 bpm | -0.0137890 | 0.0155245 | 0.374 | 0.610 |
|  | RHR 70-79 bpm | -0.0147655 | 0.0144799 | 0.308 | 0.610 |
|  | RHR >80bpm | -0.0388716 | 0.0182809 | 0.033 | 0.236 |
| CA4-body | RHR <60 bpm | 0.0019261 | 0.0156855 | 0.902 | 0.933 |
|  | RHR 70-79 bpm | 0.0093161 | 0.0146341 | 0.524 | 0.707 |
|  | RHR >80bpm | 0.0046574 | 0.0184787 | 0.801 | 0.874 |
| CA4-head | RHR <60 bpm | -0.0203930 | 0.0152227 | 0.180 | 0.590 |
|  | RHR 70-79 bpm | -0.0050655 | 0.0142050 | 0.721 | 0.833 |
|  | RHR >80bpm | -0.0227717 | 0.0179238 | 0.204 | 0.597 |
| Fimbria | RHR <60 bpm | 0.0019219 | 0.0156398 | 0.902 | 0.933 |
|  | RHR 70-79 bpm | -0.0402193 | 0.0145921 | 0.006 | 0.070 |
|  | RHR >80bpm | -0.0704091 | 0.0184226 | <0.001 | 0.004 |
| GC-ML-DG-body | RHR <60 bpm | 0.0081824 | 0.0153889 | 0.595 | 0.751 |
|  | RHR 70-79 bpm | 0.0024503 | 0.0143569 | 0.864 | 0.925 |
|  | RHR >80bpm | -0.0098363 | 0.0181264 | 0.587 | 0.750 |
| GC-ML-DG-head | RHR <60 bpm | -0.0222215 | 0.0152544 | 0.145 | 0.540 |
|  | RHR 70-79 bpm | -0.0128272 | 0.0142321 | 0.367 | 0.610 |
|  | RHR >80bpm | -0.0355173 | 0.0179633 | 0.048 | 0.320 |
| Hata | RHR <60 bpm | 0.0064412 | 0.0156964 | 0.682 | 0.818 |
|  | RHR 70-79 bpm | -0.0190041 | 0.0146425 | 0.194 | 0.597 |
|  | RHR >80bpm | -0.0299087 | 0.0184841 | 0.106 | 0.423 |
| Hippocampal-fissure | RHR <60 bpm | -0.0073495 | 0.0152959 | 0.631 | 0.780 |
|  | RHR 70-79 bpm | 0.0315536 | 0.0142692 | 0.027 | 0.219 |
|  | RHR >80bpm | 0.0466409 | 0.0180358 | 0.010 | 0.106 |
| Hippocampal-tail | RHR <60 bpm | -0.0219537 | 0.0153587 | 0.153 | 0.540 |
|  | RHR 70-79 bpm | 0.0039530 | 0.0143268 | 0.783 | 0.870 |
|  | RHR >80bpm | 0.0140976 | 0.0180907 | 0.436 | 0.654 |
| Paralaminar-nucleus | RHR <60 bpm | -0.0255584 | 0.0149265 | 0.087 | 0.379 |
|  | RHR 70-79 bpm | -<0.0011951 | 0.0139289 | 0.989 | 0.989 |
|  | RHR >80bpm | -0.0158262 | 0.0175944 | 0.368 | 0.610 |
| Presubiculum-body | RHR <60 bpm | -0.0183907 | 0.0151615 | 0.225 | 0.600 |
|  | RHR 70-79 bpm | -0.0137567 | 0.0141435 | 0.331 | 0.610 |
|  | RHR >80bpm | 0.0186141 | 0.0178643 | 0.297 | 0.610 |
| Presubiculum-head | RHR <60 bpm | -0.0148955 | 0.0152909 | 0.330 | 0.610 |
|  | RHR 70-79 bpm | -0.0108254 | 0.0142662 | 0.448 | 0.658 |
|  | RHR >80bpm | <0.0013162 | 0.0180103 | 0.986 | 0.989 |
| Subiculum-body | RHR <60 bpm | -0.0125686 | 0.0154698 | 0.417 | 0.643 |
|  | RHR 70-79 bpm | -0.0025404 | 0.0144272 | 0.860 | 0.925 |
|  | RHR >80bpm | -0.0057103 | 0.0182173 | 0.754 | 0.854 |
| Subiculum-head | RHR <60 bpm | -0.0192992 | 0.0155500 | 0.215 | 0.599 |
|  | RHR 70-79 bpm | -0.0133015 | 0.0144981 | 0.359 | 0.610 |
|  | RHR >80bpm | -0.0077606 | 0.0183004 | 0.672 | 0.818 |
| Whole-hippocampal-body | RHR <60 bpm | -0.0115761 | 0.0148244 | 0.435 | 0.654 |
|  | RHR 70-79 bpm | -0.0050650 | 0.0138296 | 0.714 | 0.833 |
|  | RHR >80bpm | -0.0189936 | 0.0174650 | 0.277 | 0.610 |
| Whole-hippocampal-head | RHR <60 bpm | -0.0139871 | 0.0147320 | 0.342 | 0.610 |
|  | RHR 70-79 bpm | -0.0085838 | 0.0137461 | 0.532 | 0.710 |
|  | RHR >80bpm | -0.0148585 | 0.0173458 | 0.392 | 0.618 |
| Whole-hippocampus | RHR <60 bpm | -0.0195546 | 0.0145417 | 0.179 | 0.590 |
|  | RHR 70-79 bpm | -0.0020693 | 0.0135593 | 0.879 | 0.925 |
|  | RHR >80bpm | -0.0115070 | 0.0171140 | 0.501 | 0.695 |
| Abbreviation: RHR, resting heart rate  *RHR 60-69 bpm is set as reference | | | | | |

## The associations between continuous measures of resting heart rate and fractional anisotropy values of white matter tracts

| **SM 7 Table 3 - The associations between continuous measures of resting heart rate and fractional anisotropy values of white matter tracts** | | | | |
| --- | --- | --- | --- | --- |
| **White matter microstructure** | **β - coefficients** | **Standard Error** | ***P*** | **FDR - *P*** |
| **Left tracts** |  |  |  |  |
| Medial lemniscus | <0.0010693 | <0.0010142 | <0.001 | <0.001 |
| Posterior thalamic radiation | -<0.0010555 | <0.0010123 | <0.001 | <0.001 |
| Inferior fronto-occipital fasciculus | -<0.0010401 | <0.0010126 | 0.001 | 0.008 |
| Anterior thalamic radiation | -<0.0010199 | <0.0010106 | 0.061 | 0.150 |
| Inferior longitudinal fasciculus | -<0.0010219 | <0.0010117 | 0.061 | 0.150 |
| Superior thalamic radiation | <0.0010218 | <0.0010110 | 0.047 | 0.150 |
| Parahippocampal part of cingulum | <0.0010288 | <0.0010165 | 0.080 | 0.167 |
| Acoustic radiation | <0.0010150 | <0.0010127 | 0.239 | 0.389 |
| Superior longitudinal fasciculus | -<0.0010134 | <0.0010119 | 0.259 | 0.389 |
| Corticospinal tract | -<0.0010059 | <0.0010132 | 0.655 | 0.684 |
| Uncinate fasciculus | <0.0010059 | <0.0010133 | 0.658 | 0.684 |
| Cingulate gyrus part of cingulum | -<0.0010003 | <0.0010198 | 0.988 | 0.988 |
| **Right tracts** |  |  |  |  |
| Acoustic radiation | <0.0010540 | <0.0010122 | <0.001 | <0.001 |
| Parahippocampal part of cingulum | <0.0010595 | <0.0010175 | 0.001 | 0.005 |
| Posterior thalamic radiation | -<0.0010349 | <0.0010118 | 0.003 | 0.015 |
| Anterior thalamic radiation | -<0.0010200 | <0.0010104 | 0.056 | 0.150 |
| Cingulate gyrus part of cingulum | <0.0010340 | <0.0010194 | 0.080 | 0.167 |
| Medial lemniscus | <0.0010245 | <0.0010144 | 0.089 | 0.172 |
| Corticospinal tract | -<0.0010208 | <0.0010135 | 0.123 | 0.221 |
| Inferior fronto-occipital fasciculus | -<0.0010137 | <0.0010119 | 0.249 | 0.389 |
| Inferior longitudinal fasciculus | -<0.0010109 | <0.0010110 | 0.322 | 0.425 |
| Uncinate fasciculus | <0.0010112 | <0.0010119 | 0.347 | 0.426 |
| Superior thalamic radiation | <0.0010097 | <0.0010111 | 0.382 | 0.448 |
| Superior longitudinal fasciculus | -<0.0010086 | <0.0010117 | 0.460 | 0.517 |
| **Other tract** |  |  |  |  |
| Forceps minor | -<0.0010235 | <0.0010118 | 0.047 | 0.150 |
| Middle cerebellar peduncle | <0.0010164 | <0.0010156 | 0.295 | 0.419 |
| Forceps major | -<0.0010151 | <0.0010155 | 0.330 | 0.425 |

## The associations between categorical measures of resting heart rate and fractional anisotropy values of white matter tracts

| **SM 7 Table 4 - The associations between categorical measures of resting heart rate and fractional anisotropy values of white matter tracts** | | | | |
| --- | --- | --- | --- | --- |
| **White matter microstructure** | **RHR** | **β - coefficients** | **Standard error** | ***P*** |
| **Left tracts** |  |  |  |  |
| Acoustic radiation | RHR <60 bpm | -<0.0017426 | <0.0013632 | 0.041 |
|  | RHR 70-79 bpm | -<0.0010625 | <0.0013379 | 0.853 |
|  | RHR >80bpm | <0.0010095 | <0.0014250 | 0.982 |
| Anterior thalamic radiation | RHR <60 bpm | -<0.0010882 | <0.0013025 | 0.771 |
|  | RHR 70-79 bpm | -<0.0011672 | <0.0012815 | 0.553 |
|  | RHR >80bpm | -<0.0018599 | <0.0013544 | 0.015 |
| Cingulate gyrus part of cingulum | RHR <60 bpm | -<0.0011298 | <0.0015640 | 0.818 |
|  | RHR 70-79 bpm | <0.0011143 | <0.0015248 | 0.828 |
|  | RHR >80bpm | -<0.0013377 | <0.0016604 | 0.609 |
| Parahippocampal part of cingulum | RHR <60 bpm | -<0.0014491 | <0.0014702 | 0.340 |
|  | RHR 70-79 bpm | -<0.0014271 | <0.0014373 | 0.329 |
|  | RHR >80bpm | <0.0016681 | <0.0015501 | 0.224 |
| Corticospinal tract | RHR <60 bpm | <0.0011060 | <0.0013757 | 0.778 |
|  | RHR 70-79 bpm | <0.0012851 | <0.0013495 | 0.415 |
|  | RHR >80bpm | -<0.0012771 | <0.0014398 | 0.529 |
| Inferior fronto-occipital fasciculus | RHR <60 bpm | -<0.0010135 | <0.0013587 | 0.970 |
|  | RHR 70-79 bpm | -<0.0015720 | <0.0013336 | 0.086 |
|  | RHR >80bpm | -0.0013651 | <0.0014200 | 0.001 |
| Inferior longitudinal fasciculus | RHR <60 bpm | -<0.0011812 | <0.0013337 | 0.587 |
|  | RHR 70-79 bpm | -<0.0013902 | <0.0013102 | 0.208 |
|  | RHR >80bpm | -<0.0019591 | <0.0013905 | 0.014 |
| Medial lemniscus | RHR <60 bpm | -<0.0016681 | <0.0014040 | 0.098 |
|  | RHR 70-79 bpm | <0.0018128 | <0.0013759 | 0.031 |
|  | RHR >80bpm | 0.0011977 | <0.0014729 | 0.011 |
| Posterior thalamic radiation | RHR <60 bpm | <0.0013475 | <0.0013512 | 0.322 |
|  | RHR 70-79 bpm | -<0.0017274 | <0.0013266 | 0.026 |
|  | RHR >80bpm | -0.0014953 | <0.0014115 | <0.001 |
| Superior longitudinal fasciculus | RHR <60 bpm | -<0.0012276 | <0.0013385 | 0.501 |
|  | RHR 70-79 bpm | -<0.0012643 | <0.0013148 | 0.401 |
|  | RHR >80bpm | -<0.0015373 | <0.0013967 | 0.176 |
| Superior thalamic radiation | RHR <60 bpm | -<0.0015049 | <0.0013123 | 0.106 |
|  | RHR 70-79 bpm | <0.0011725 | <0.0012908 | 0.553 |
|  | RHR >80bpm | <0.0011264 | <0.0013658 | 0.730 |
| Uncinate fasciculus | RHR <60 bpm | -<0.0012823 | <0.0013804 | 0.458 |
|  | RHR 70-79 bpm | -<0.0011982 | <0.0013539 | 0.575 |
|  | RHR >80bpm | -<0.0012780 | <0.0014455 | 0.533 |
| **Right tracts** |  |  |  |  |
| Acoustic radiation | RHR <60 bpm | -<0.0016631 | <0.0013487 | 0.057 |
|  | RHR 70-79 bpm | <0.0017978 | <0.0013246 | 0.014 |
|  | RHR >80bpm | <0.0019643 | <0.0014085 | 0.018 |
| Anterior thalamic radiation | RHR <60 bpm | -<0.0010586 | <0.0012978 | 0.844 |
|  | RHR 70-79 bpm | -<0.0010964 | <0.0012770 | 0.728 |
|  | RHR >80bpm | -<0.0017546 | <0.0013488 | 0.031 |
| Cingulate gyrus part of cingulum | RHR <60 bpm | -0.0011536 | <0.0015542 | 0.037 |
|  | RHR 70-79 bpm | <0.0010575 | <0.0015156 | 0.911 |
|  | RHR >80bpm | <0.0010670 | <0.0016491 | 0.918 |
| Parahippocampal part of cingulum | RHR <60 bpm | -<0.0015121 | <0.0015008 | 0.306 |
|  | RHR 70-79 bpm | <0.0010675 | <0.0014661 | 0.885 |
|  | RHR >80bpm | 0.0014355 | <0.0015862 | 0.014 |
| Corticospinal tract | RHR <60 bpm | <0.0012616 | <0.0013844 | 0.496 |
|  | RHR 70-79 bpm | <0.0010742 | <0.0013577 | 0.836 |
|  | RHR >80bpm | -<0.0015635 | <0.0014502 | 0.211 |
| Inferior fronto-occipital fasciculus | RHR <60 bpm | -<0.0011152 | <0.0013394 | 0.734 |
|  | RHR 70-79 bpm | -<0.0011253 | <0.0013157 | 0.691 |
|  | RHR >80bpm | -<0.0015002 | <0.0013975 | 0.208 |
| Inferior longitudinal fasciculus | RHR <60 bpm | <0.0010622 | <0.0013143 | 0.843 |
|  | RHR 70-79 bpm | -<0.0010201 | <0.0012923 | 0.945 |
|  | RHR >80bpm | -<0.0013770 | <0.0013681 | 0.306 |
| Medial lemniscus | RHR <60 bpm | -<0.0013257 | <0.0014110 | 0.428 |
|  | RHR 70-79 bpm | <0.0011879 | <0.0013824 | 0.623 |
|  | RHR >80bpm | <0.0013799 | <0.0014818 | 0.430 |
| Posterior thalamic radiation | RHR <60 bpm | <0.0014161 | <0.0013373 | 0.217 |
|  | RHR 70-79 bpm | -<0.0011398 | <0.0013138 | 0.656 |
|  | RHR >80bpm | -<0.0018429 | <0.0013951 | 0.033 |
| Superior longitudinal fasciculus | RHR <60 bpm | -<0.0010969 | <0.0013325 | 0.771 |
|  | RHR 70-79 bpm | -<0.0011030 | <0.0013093 | 0.739 |
|  | RHR >80bpm | -<0.0013419 | <0.0013896 | 0.380 |
| Superior thalamic radiation | RHR <60 bpm | -<0.0013432 | <0.0013159 | 0.277 |
|  | RHR 70-79 bpm | <0.0012109 | <0.0012939 | 0.473 |
|  | RHR >80bpm | -<0.0010919 | <0.0013699 | 0.804 |
| Uncinate fasciculus | RHR <60 bpm | -<0.0015938 | <0.0013401 | 0.081 |
|  | RHR 70-79 bpm | <0.0011009 | <0.0013164 | 0.750 |
|  | RHR >80bpm | -<0.0014043 | <0.0013982 | 0.310 |
| **Other tracts** |  |  |  |  |
| Forceps major | RHR <60 bpm | <0.0014991 | <0.0014419 | 0.259 |
|  | RHR 70-79 bpm | <0.0012523 | <0.0014111 | 0.539 |
|  | RHR >80bpm | -<0.0015000 | <0.0015176 | 0.334 |
| Forceps minor | RHR <60 bpm | -<0.0010403 | <0.0013368 | 0.905 |
|  | RHR 70-79 bpm | -<0.0013266 | <0.0013133 | 0.297 |
|  | RHR >80bpm | -<0.0017227 | <0.0013944 | 0.067 |
| Middle cerebellar peduncle | RHR <60 bpm | -<0.0012224 | <0.0014452 | 0.617 |
|  | RHR 70-79 bpm | <0.0016021 | <0.0014146 | 0.146 |
|  | RHR >80bpm | <0.0013955 | <0.0015216 | 0.448 |
| Abbreviation: RHR, resting heart rate  *RHR 60-69 bpm is set as reference | | | | |

# *Appendix* 1 – Field IDs and of UK Biobank

| **Variates** | **Field ID** |
| --- | --- |
| Resting heart rate | 102 |
| Age | 21022 |
| Female | 31 |
| Education | 6138 |
| Smoking status | 20116 |
| Physical activity |  |
| MET minutes per week for walking | 22037 |
| MET minutes per week for moderate activity | 22038 |
| MET minutes per week for vigorous activity | 22039 |
| BMI | 21001 |
| Total cholestrol | 23400 |
| Diabetes | 130707-130715 |
| Hypertension |  |
| Systolic blood pressure | 4080 |
| Diastolic blood pressure | 4079 |
| Heart failure | 131354, 131355 |
| Atrial fibrillation | 131350, 131351 |
| Ischemic heart disease | 131306, 131307 |
| Cerebrovascular disease | 131361-131379 |
| Use of RHR-reducing medications | 20003 |
| Prospective memory | 20018 |
| Pairs matching | 399 |
| Numeric memory | 4282 |
| Reaction time | 20023 |
| Fluid intelligence | 20016 |

# *Appendix* 2 – Catalogue of rest heart rate reduction medications in UK biobank

| **Use of RHR-reducing medications** |  |
| --- | --- |
| **Beta blockers** |  |
| carvedilol | 1140909368 |
| propranolol hydrochloride+bendrofluazide 80mg/2.5mg capsule | 1140860418 |
| atenolol+nifedipine 50mg/20mg m/r capsule | 1140860426 |
| atenolol | 1140866738 |
| oxprenolol | 1140879830 |
| propranolol | 1140879842 |
| atenolol+chlorthalidone | 1141146124 |
| atenolol+bendrofluazide | 1141146126 |
| atenolol+co-amilozide | 1141146128 |
| atenolol+chlortalidone | 1141180778 |
| atenolol+bendroflumethiazide | 1141194810 |
| metoprolol tartrate+chlorthalidone 100mg/12.5mg tablet | 1140860308 |
| metoprolol tartrate+hydrochlorothiazide 100mg/12.5mg tablet | 1140860404 |
| bisoprolol fumarate+hydrochlorothiazide 10mg/6.25mg tablet | 1140864950 |
| bisoprolol | 1140879760 |
| celiprolol | 1140879762 |
| metoprolol | 1140879818 |
| sloprolol 80mg m/r capsule | 1140916730 |
| sotalol hydrochloride+hydrochlorothiazide 80mg/12.5mg tablet | 1140860332 |
| sotalol | 1140879854 |
| nebivolol | 1141164276 |
| **Digoxin** |  |
| digoxin product | 1140865966 |
| medigoxin | 1140866058 |
| digoxin | 2038459814 |
| cardiacap 30mg m/r capsule | 1140851726 |
| cedilanid 250micrograms tablet | 1140866054 |
| digitalis | 1140910404 |
| **Non-dihydropyridine calcium channel blockers** |  |
| verapamil | 1140888510 |
| trandolapril+verapamil hydrochloride | 1141153328 |
| diltiazem | 1140879806 |
| diltiazem hcl+hydrochlorothiazide 150mg/12.5mg m/r capsule | 1140926778 |
